# Supplementary material for: Dynamics of non-self-similar earthquakes illuminated by a controlled fault asperity
Source: Nat Commun. 2026 Apr 30;17:3860. doi: 10.1038/s41467-026-72217-x (PMC13133396; doi:10.1038/s41467-026-72217-x)
Supplement: Supplementary file 1 — Supplementary Information [file 41467_2026_72217_MOESM1_ESM.pdf]

Supplementary Information for "Dynamics of non-self-similar earthquakes illuminated by a controlled fault asperity"

Kurama Okubo[1*], Futoshi Yamashita[1†] and Eiichi Fukuyama[2,1†]

[1*]National Research Institute for Earth Science and Disaster Resilience (NIED), Tsukuba, Japan.
[2]Department of Civil and Earth Resources Engineering, Kyoto University, Japan.

*Corresponding author(s). E-mail(s): kokubo@bosai.go.jp;
[†]These authors contributed equally to this work.

Contents of this file

1. Supplementary Notes S1 to S8
2. Supplementary Tables S1 to S4
3. Supplementary Figures S1 to S26

Supplementary Note S1: Spectral ratio analysis

We performed spectral ratio analysis to examine the non-self-similarity of the gouge patch (GP) events (Figs. 3d and S8). In this analysis, the AE waveforms of the GP events were used without correcting for instrumental response, sensor coupling factors, or attenuation effects. An 80 kHz fourth-order Butterworth high-pass filter was applied to remove long-period noise. Then, the P-wave window was trimmed from 1 μs before to 6 μs after the P-wave onset. The amplitude spectra of the P-wave window were computed by zero-padding to 256 data points after demeaning, detrending, and applying a 20% Tukey window. The spectral ratio was obtained by dividing the amplitude spectra of the largest event (D129) by those of the smaller events (D24, D50, D52, and D72), representing the ratio of the source spectra under the assumption that the instrumental response and path effects were canceled out. Finally, the mean spectral ratio was computed by averaging the spectral ratios obtained from the four AE sensors closest to P3.

Supplementary Note S2: Comparison of seismic moment of GP events with recorded physical parameters

We examined how the seismic moment M_0 of the GP events relates to macroscopic and local measurements of physical parameters, in order to assess whether any of these quantities provide insight into the rupture mechanisms on the GP and help explain the observed variation in M_0 (Fig. S11). Below, we describe the metrics used to quantify each parameter from the macroscopic and local measurements. Hereafter, we use the term stick–slip events to denote ruptures involving the entire laboratory fault, in contrast to ruptures confined to the GP.

S2.1: Local slip velocity

The local slip velocity for each GP event was estimated by linearly interpolating two gap-sensor records located near the GP. The slip velocity at the GP onset time was computed from this interpolation procedure, following the approach of Yamashita et al. (2022).

S2.2: Local normal stress

Although the flat-jack pressures generating the normal load are mechanically maintained at constant levels, the local normal stress surrounding the GP can be slightly perturbed during each stick–slip event due to the dynamics of preslip and main shock. To capture this effect, we evaluated the normal-stress component recorded by the triaxial strain gauges near P3 (SGT07 and SGT23) installed on the side surfaces of the rock specimen, averaging the measurements over a ± 1 ms window centered on the onset of each GP event.

S2.3: Macroscopic shear stress drop

The macroscopic shear stress was computed as the shear load measured by the load cell divided by the nominal fault area (4 m $\times$ 0.1 m). The macroscopic shear stress

drop for each stick–slip event was then defined as the difference between the shear stress immediately before and after the main shock of the stick-slip event.

S2.4: Coseismic slip of each stick–slip event

The coseismic slip of each stick–slip event was evaluated as the mean slip recorded by the 16 gap sensors. Each GP event was associated with the stick–slip event during which it occurred.

S2.5: Hold time

To investigate whether time-dependent frictional healing influences variations in the seismic moment of GP events, we analyzed the hold time, defined as the duration between the preceding stick–slip event and the onset of GP foreshocks.

S2.6: Total macroscopic slip

The total macroscopic slip was computed as the cumulative sum of the coseismic slip over successive stick–slip events, eventually reaching approximately 2 mm during the experiment. Each GP event was linked to the cumulative slip value corresponding to the stick–slip event in which it occurred.

Supplementary Note S3: Measurements of GP physical properties

S3.1: Normal pressure

To evaluate the normal stress concentration caused by the topographic gap on the GP, we investigated the pressure distribution using a pressure-sensitive film, `Prescale LW` (Fujifilm), which has a measurement range of 2.5–10 MPa. A set of cylindrical rock specimens (diameter = 25 mm) was installed in a uniaxial pressure machine, with a GP (diameter = 8 mm) placed between them.

We conducted a uniaxial loading test following the manufacturer's instructions for `Prescale`. A two-sheet type pressure-sensitive film was inserted at the GP interface. Normal loading was then applied and increased linearly to 2.0 MPa over a two-minute period, after which it was held constant for an additional two minutes. The load was subsequently released, and the resulting color distribution on the film was analyzed.

To convert the color density (Fig. S15a) into pressure values (Fig. S15b), we used the `Prescale Mobile` app. The discolored film was scanned using an iPad Pro 11-inch (1st generation), placed on a color calibration sheet specifically designed for pressure scanning with `Prescale Mobile`.

S3.2: Topography

We placed the GP on a 40-mm cubic rock specimen fixed on a servo-controlled moving stage. The surface height was continuously measured using a laser displacement transducer (LT-9010M, Keyence; minimum vertical resolution: ∼10 nm) while the stage was moved. From these measurements, we obtained the two-dimensional topography of the GP, as shown in Fig. S15c, with a horizontal resolution of 50 μm.

Supplementary Note S4: Least-squares method for estimating the ARX model of the AE sensor

We review the auto-regressive model with exogenous input (ARX), following Ljung (1987), to derive the least-squares estimation of the model parameters. The ARX model is formulated as follows:

$$y_k = \frac{B(q)}{A(q)} u_k + \frac{1}{A(q)} e_k, \tag{S1}$$

where u_k and y_k are the discrete time series of the input and output on the system, corresponding to the LDV and AE sensor measurements in this study, respectively. The time index k is defined such that $t = (k-1)\Delta t$, where Δt is the data time step. The term q is the time shift operator, satisfying $q^{-1} y_k = y_{k-1}$, and e_k denotes white noise. The polynomials $A(q)$ and $B(q)$ are defined as:

$$\begin{aligned} A(q) &= 1 + a_1 q^{-1} + \cdots + a_m q^{-m} \\ B(q) &= b_0 + b_1 q^{-1} + \cdots + b_n q^{-n}, \end{aligned}$$

where m and n are the numbers of poles and zeros, respectively (see also SEED Reference Manual, 2012, Appendix C). Note that we include b_0 as a model parameter, following McLaskey and Glaser (2012). To handle negative time values, we padded zero to the input and output data accordingly.

The one-step predictor for Equation (S1) is given by:

$$\hat{y}_k(\boldsymbol{\theta}) = \boldsymbol{\phi}_k^T \boldsymbol{\theta}, \tag{S2}$$

where $\boldsymbol{\theta}$ indicates the vector of model parameters:

$$\boldsymbol{\theta} = [a_1, a_2, \cdots, a_m, b_0, b_1, \cdots, b_n]^T, \tag{S3}$$

and $\boldsymbol{\phi}_k$ is a column vector of inputs and outputs:

$$\boldsymbol{\phi}_k = [-y_{k-1}, -y_{k-2}, \cdots, -y_{k-m}, u_k, u_{k-1}, \cdots, u_{k-n}]^T. \tag{S4}$$

The prediction error $\varepsilon_k(\boldsymbol{\theta})$ is defined as:

$$\varepsilon_k(\boldsymbol{\theta}) = y_k - \boldsymbol{\phi}_k^T \boldsymbol{\theta}. \tag{S5}$$

The cost function $\ell(\boldsymbol{\theta})$ is formulated as:

$$\ell(\boldsymbol{\theta}) = \frac{1}{N} \sum_{k=1}^{N} \frac{1}{2} [y_k - \boldsymbol{\phi}_k^T \boldsymbol{\theta}]^2, \tag{S6}$$

where N is the number of input and output data points.

Following the Problem 7D.2 of Ljung (1987), we expand the cost function as:

$$\begin{aligned}\ell(\boldsymbol{\theta}) &= \frac{1}{N}\sum_{k=1}^{N}\frac{1}{2}[y_k - \boldsymbol{\phi}_k^T\boldsymbol{\theta}]^2 \\ &= \boldsymbol{\theta}^T A\boldsymbol{\theta} - \boldsymbol{\theta}^T B - B^T\boldsymbol{\theta} + C,\end{aligned} \tag{S7}$$

where

$$A = \frac{1}{N}\sum_{k=1}^{N}\boldsymbol{\phi}_k\boldsymbol{\phi}_k^T,$$

$$B = \frac{1}{N}\sum_{k=1}^{N}\boldsymbol{\phi}_k y_k,$$

$$C = \frac{1}{N}\sum_{k=1}^{N}y_k^2.$$

The right-hand side of Equation (S7) can be rewritten as

$$\left[\boldsymbol{\theta} - A^{-1}B\right]^T A\left[\boldsymbol{\theta} - A^{-1}B\right] + C - B^T A^{-1}B. \tag{S8}$$

Since A is a positive symmetric semidefinite matrix, Equation (S8) is minimized when:

$$\boldsymbol{\theta} - A^{-1}B = \mathbf{0}, \tag{S9}$$

which results in the least-squares estimate of the model parameters:

$$\hat{\boldsymbol{\theta}} = \left[\frac{1}{N}\sum_{k=1}^{N}\boldsymbol{\phi}_k\boldsymbol{\phi}_k^T\right]^{-1}\frac{1}{N}\sum_{k=1}^{N}\boldsymbol{\phi}_k y_k. \tag{S10}$$

Supplementary Note S5: Modeling waveform propagation generated by ball-drop impact

We modeled waveform propagation in a 2.0 m segment of the bottom rock specimen, applying a perfectly matched layer (PML) absorbing boundaries on the longitudinal side surfaces. The velocity model consisted of the rock specimen with a uniform velocity structure (Table S4) with a 20 mm thick steel base plate with elastic properties of $\rho = 7.85$ g/cm^3, $c_p = 5.9$ km/s, $c_s = 3.23$ km/s, as referenced in McLaskey and Glaser (2010). A uniform grid spacing of 0.5 mm was set to achieve a resolution of more than 10 grid points per wavelength at 600 kHz. The force-time function of the ball-drop source was derived following the formulation of McLaskey and Glaser (2010). To efficiently compute the Green's function, we calculated the relative positions of the AE sensor and ball-drop source and used the reciprocity mode in `OpenSWPC`, allowing the simulation to be completed in a single run.

Supplementary Note S6: Method to constrain the sensor coupling factor using ball-drop impact

The residual of the P-wave amplitude used to optimize the sensor coupling factor is defined as:

$$\min \; \|A_{ij}^{\mathrm{obs}} - \hat{A}_{ij}\|, \tag{S11}$$

where A_{ij}^{obs} is the amplitude of the observed waveform, corrected for the instrumental response, recorded by the i-th AE sensor from the j-th ball-drop source. The modeled amplitude $\hat{A}_{ij}$ is expressed as:

$$\hat{A}_{ij} = A_{ij}^{\mathrm{model}} S_i T_j \beta(\omega, \theta), \tag{S12}$$

where A_{ij}^{model} is the P-wave amplitude of the simulated waveform, S_i is the sensor coupling factor for the ith AE sensor, T_j is a correction factor accounting for variations in the ball-drop impact at the j-th source, and $\beta(\omega, \theta)$ is the aperture effect factor (Miller and McIntire, 1987; McLaskey and Glaser, 2012), as defined in Equation (2) of the main text. To account for variability in the impact amplitude on the fault surface, we incorporated the factor T_j into the amplitude model proposed by Kwiatek et al. (2014). This compensates for deviations from the estimates based on Hertzian contact theory, which may arise due to variations in the fault surface state. We assumed a constant quality factor of $Q_p^{\mathrm{const}} = 200$ in the simulated waveforms to compute A_{ij}^{model}, as described in the Methods section of the main text. The P-wave amplitudes for both observed and modeled waveforms were determined as the maximum value within the P-wave window of the vertical velocity component on the side surface of the rock specimen after applying a 10–400 kHz band-pass filter.

We excluded the source-station pairs with a source distance greater than 400 mm. We also computed the arrival time difference between the direct P-wave and the reflected P-wave from the side surface of the rock specimen. Pairs with an arrival time difference smaller than 6 μs were excluded to avoid potential bias from variations in the incident angles of the reflected wave. As a result, 117 source-station pairs were used to constrain the amplitude factors S_i, T_j, and the angular frequency ω of the incident wave. The model parameters in Equation (S12) were optimized using the `lsqnonlin` function in the `MATLAB Optimization Toolbox`, with the `trust-region-reflective` algorithm.

Supplementary Note S7: Derivation of aperture effect for arbitrary incident angle

We derive the aperture effect factor for an arbitrary incident angle, extending the formulation originally developed for Rayleigh waves (incident angle $\theta = \pi/2$) by Miller and McIntire (1987, p. 128, Equation 4). The sensor response is evaluated as the average local amplitude over the sensor surface, given by the following equation:

$$y(t, \theta) = \frac{1}{\pi R^2} \int_0^{2\pi} \int_0^{R} u(t, \theta, r, \phi) r \, dr \, d\phi, \tag{S13}$$

where $y(t, \theta)$ is an output of the sensor, θ is an incident angle of a given plane wave, R is the sensor radius, and $u(t, \theta, r, \phi)$ is the local motion at the polar coordinate (r, ϕ) on the sensor surface (Fig. S24). Note that u can represent either displacement or velocity, depending on the characteristics of the AE sensor response.

The time difference Δt in wave arrival between the center of the sensor and a local position (r, ϕ) on the sensor surface is given by:

$$\Delta t = \frac{r \cos\phi \sin\theta}{v} = \frac{r\cos\phi}{v_a}, \tag{S14}$$

where v is the wave speed in the medium and $v_a = v/\sin\theta$ is the apparent velocity of the wavefront projected along the sensor surface.

Assuming that the incident wave is a cosine wave with angular frequency ω, the sensor response can be evaluated as the spatial average of the local motion over the sensor surface. The local motion at a point on the surface is expressed as:

$$u(t, \omega, \theta, r, \phi) = \cos\left[\omega\left(t + r\cos\phi/v_a\right)\right], \tag{S15}$$

Thus, the sensor surface response becomes:

$$y(t, \omega, \theta) = \frac{1}{\pi R^2}\int_0^{2\pi}\int_0^{R} \cos\left[\omega\left(t + r\cos\phi/v_a\right)\right] r\, dr\, d\phi. \tag{S16}$$

The next step is to evaluate the integral in Equation (S16) using the following formulas:

1.
$$J_0(z) = \frac{1}{2\pi}\int_0^{2\pi} e^{iz\cos\phi}\, d\phi \tag{S17}$$

2.
$$\int azJ_0(az)\, dz = zJ_1(az), \tag{S18}$$

where $J_0(z)$ and $J_1(z)$ are the Bessel functions of the first kind. Equation (S17) can be derived from Watson (1944, section 2.2 Equation 5) by setting $\alpha = -\pi/2$. Equation (S18) is obtained from Watson (1944, section 2.1.2, Equations 3 and 5) by replacing z to az and performing the integral.

We rewrite Equation (S16) as follows:

$$y(t, \omega, \theta) = \frac{1}{\pi R^2}\Re\left[\int_0^{2\pi}\int_0^{R} e^{i\omega(t + r\cos\phi/v_a)} r\, dr\, d\phi\right]. \tag{S19}$$

The integral can be simplified using the formula from Equations (S17) and (S18) as follows:

$$\int_0^{2\pi}\int_0^{R} e^{i\omega(t + r\cos\phi/v_a)} r\, dr\, d\phi = \frac{2\pi R v_a}{\omega} e^{i\omega t} J_1\left(\frac{\omega R}{v_a}\right). \tag{S20}$$

Thus, the sensor response y with the aperture effect factor is given by:

$$y(t,\omega,\theta) = \frac{2v_a}{\omega R} J_1\left(\frac{\omega R}{v_a}\right)\cos(\omega t). \quad \text{(S21)}$$

The coefficient of $\cos(\omega t)$ is used as the amplitude correction factor for the aperture effect. Note that we define this factor as unity for $\theta = 0$. This amplitude factor corresponds to the one proposed by Miller and McIntire (1987, Equation 4) for surface waves, which is given by:

$$y(t,\omega,\frac{\pi}{2}) = \frac{2J_1(kR)}{kR}\cos(\omega t), \quad \text{(S22)}$$

where $k = \omega/v$.

Supplementary Note S8: Configurations for dynamic rupture modeling using UGUCA

We assumed a uniform, linear elastic medium with the elastic properties listed in Table S4. To minimize periodic boundary artifacts, the computational domain was set to 40 mm × 40 mm, which is ten times the radius of the circular source patch (PCH). The grid resolution was set to 0.04 mm, ensuring at least 20 grid points per critical nucleation radius (R_c) for all target events. The time step was 3.3 ns, and the total simulation time was 6 μs. All simulations were performed on the NIED HPC cluster, with each case executed using 96 cores in parallel.

Table S1 Observed source parameters for 33 GP events, after correction for attenuation. The standard deviation (σ^{obs}) and standard error (SE^{obs}) are calculated from the four AE sensors used in the source parameter estimation. The event type column classifies events as foreshocks (F) or aftershocks (A) based on their timing relative to the onset of the main stick–slip event.

	M_0^{obs} [Nm]	T_w^{obs} [μs]	$\sigma_{M_0}^{\mathrm{obs}}$ [Nm]	$\sigma_{T_w}^{\mathrm{obs}}$ [μs]	$SE_{M_0}^{\mathrm{obs}}$ [Nm]	$SE_{T_w}^{\mathrm{obs}}$ [μs]	Event type
D4	0.594	2.558	0.179	0.152	0.090	0.076	A
D9	0.047	2.347	0.007	0.363	0.004	0.182	F
D18	0.758	2.865	0.194	0.311	0.097	0.155	F
D19	0.056	2.485	0.017	0.270	0.009	0.135	F
D20	0.867	2.767	0.210	0.138	0.105	0.069	F
D21	0.022	2.336	0.003	0.206	0.002	0.103	F
D24	0.066	2.462	0.021	0.283	0.011	0.141	F
D27	0.764	2.553	0.211	0.202	0.105	0.101	F
D31	0.975	2.584	0.263	0.216	0.131	0.108	F
D38	0.272	2.251	0.094	0.229	0.047	0.114	F
D40	0.204	2.161	0.039	0.199	0.020	0.100	F
D43	0.187	2.363	0.048	0.187	0.024	0.093	A
D44	0.070	2.448	0.016	0.330	0.008	0.165	F
D50	0.327	2.577	0.151	0.308	0.076	0.154	F
D52	0.482	2.601	0.125	0.206	0.062	0.103	A
D61	0.741	2.614	0.193	0.198	0.097	0.099	F
D62	0.591	2.539	0.165	0.176	0.083	0.088	A
D69	0.259	2.112	0.033	0.177	0.017	0.089	A
D72	0.811	2.605	0.182	0.173	0.091	0.087	F
D77	0.778	2.631	0.357	0.185	0.178	0.092	A
D85	0.485	2.292	0.133	0.205	0.066	0.103	F
D88	1.259	2.414	0.646	0.231	0.323	0.115	F
D89	0.492	2.475	0.130	0.156	0.065	0.078	A
D95	0.912	2.775	0.268	0.118	0.134	0.059	F
D99	0.826	2.950	0.244	0.232	0.122	0.116	F
D100	0.698	2.913	0.345	0.449	0.173	0.225	A
D109	0.697	2.794	0.303	0.186	0.151	0.093	A
D118	0.076	2.001	0.010	0.219	0.005	0.110	A
D120	0.779	2.757	0.352	0.245	0.176	0.122	A
D126	0.017	1.991	0.003	0.295	0.001	0.148	F
D128	0.013	2.234	0.002	0.232	0.001	0.116	A
D129	1.134	2.626	0.407	0.173	0.204	0.087	F
D131	0.719	2.495	0.243	0.082	0.121	0.041	F

Table S2 Constrained parameters for the dynamic rupture models of non-self-similar GP target events.

	σ_n^{PCH} [MPa]	$\Delta\sigma_{\mathrm{ref}}$ [MPa]	$\bar{u}_0$ [μm]	c -	s -	τ_0^{PCH} [MPa]	$\Delta\tau$ [MPa]	μ_s -	μ_d -	D_c [μm]	D_s [μm]	G_{IIC} [J/m^2]	R_c [mm]
D24	6.0	0.42	0.03	0.980	0.695	2.09	0.29	0.36	0.3	0.013	0.071	2.18×10^{-3}	1.63
D50	6.0	2.11	0.16	0.925	0.495	2.84	1.04	0.51	0.3	0.034	0.186	2.16×10^{-2}	1.13
D52	6.0	3.11	0.24	0.925	0.460	3.23	1.43	0.58	0.3	0.043	0.235	3.62×10^{-2}	1.08
D72	6.0	5.22	0.40	0.925	0.430	4.05	2.25	0.73	0.3	0.058	0.321	7.52×10^{-2}	0.97
D129	6.0	7.31	0.56	0.925	0.410	4.80	3.00	0.86	0.3	0.071	0.393	1.21×10^{-1}	0.90

Table S3 Source parameters for the optimized dynamic rupture models, estimated by fitting the cosine STF.

	M_0^{model} [Nm]	T_w^{model} [μs]
D24	0.067	2.466
D50	0.309	2.568
D52	0.466	2.592
D72	0.822	2.613
D129	1.149	2.634

Table S4 Elastic modulus of the rock specimen (metagabbro) used in waveform propagation modeling and dynamic rupture simulations.

ρ [g/cm^3]	c_p [km/s]	c_s [km/s]	E [GPa]	G [GPa]	ν -
2.98*1	6.2*2	3.6*2	96	39	0.246

*1 From Fukuyama et al. (2016).
*2 Estimated based on the waveform speeds manually optimized during the relocation of GP events.

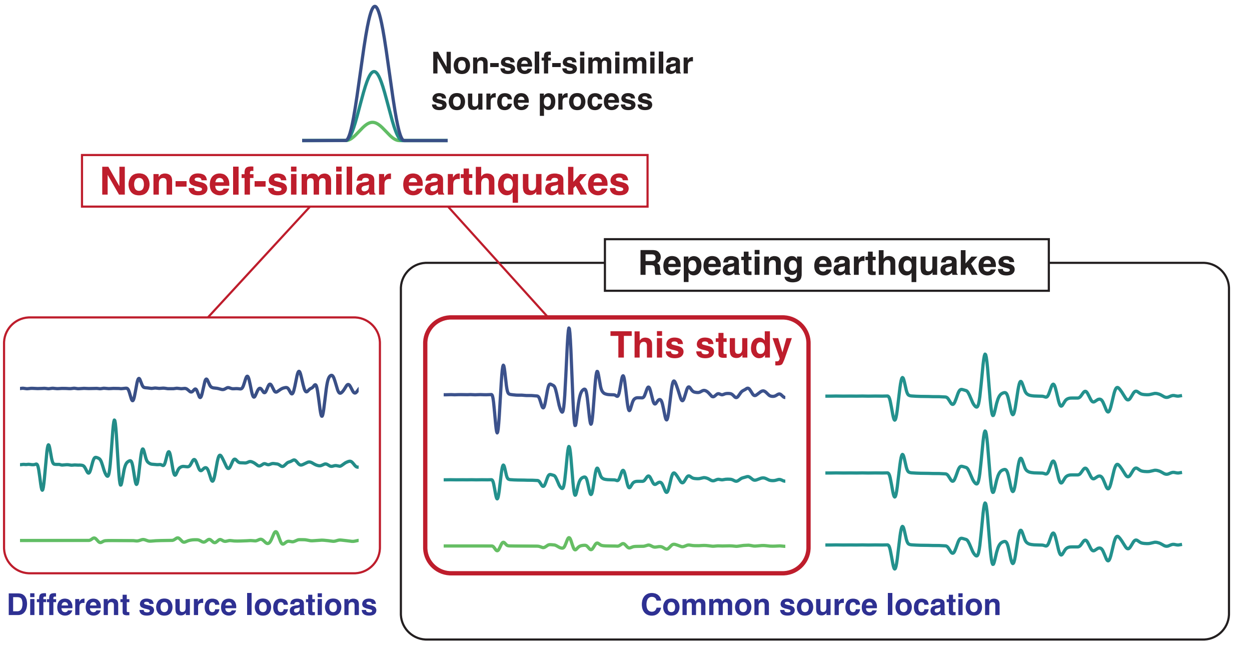

Fig. S1 Schematic illustration of the classification of non-self-similar earthquakes. Non-self-similar earthquakes are defined as events generated by a non-self-similar source process. This study focuses on clusters of non-self-similar earthquakes that share a common source location, and therefore exhibit high waveform coherence. Within this framework, repeating earthquakes are characterized by a common source location and include both non-self-similar clusters and complementary clusters exhibiting nearly identical waveforms in both phase and amplitude.

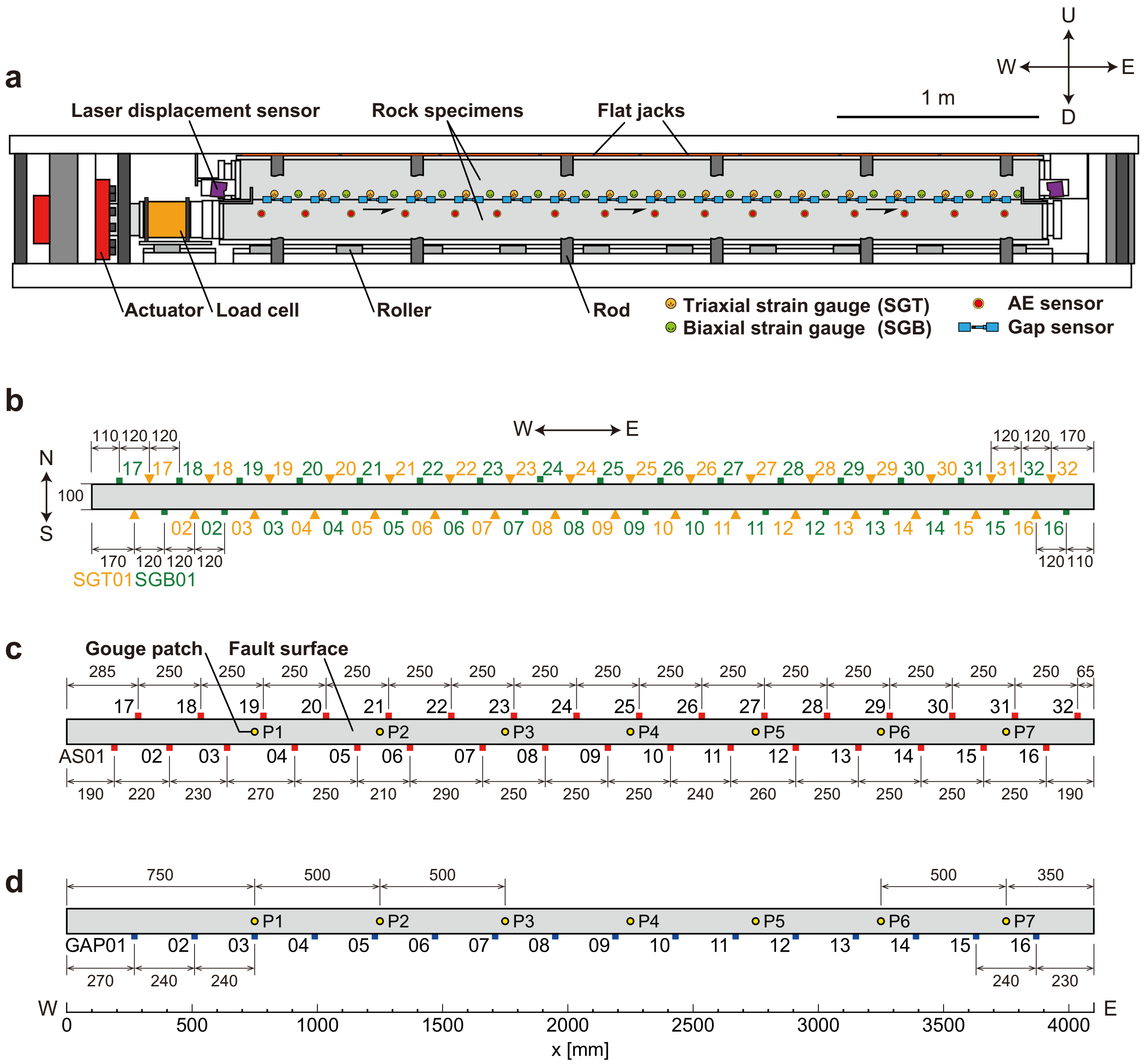

Fig. S2 Sensor arrays for local measurements. **a**, Schematic of the upper and lower rock specimens vertically stacked within the outer frames. Normal pressure and shear loading are applied using eight flat jacks and a horizontal actuator, respectively. The upper rock specimen is fixed to the frame, while the lower specimen moves from west to east. **b**, Top view of the sensor arrays, including biaxial (SGB) and triaxial (SGT) strain gauges. **c**, Acoustic emission (AE) sensors. **d**, Gap sensors used to measure the relative displacement (i.e., fault slip) across the simulated fault. Yellow circles in **c** and **d** indicate the locations of gouge patches, labeled with their respective IDs.

a

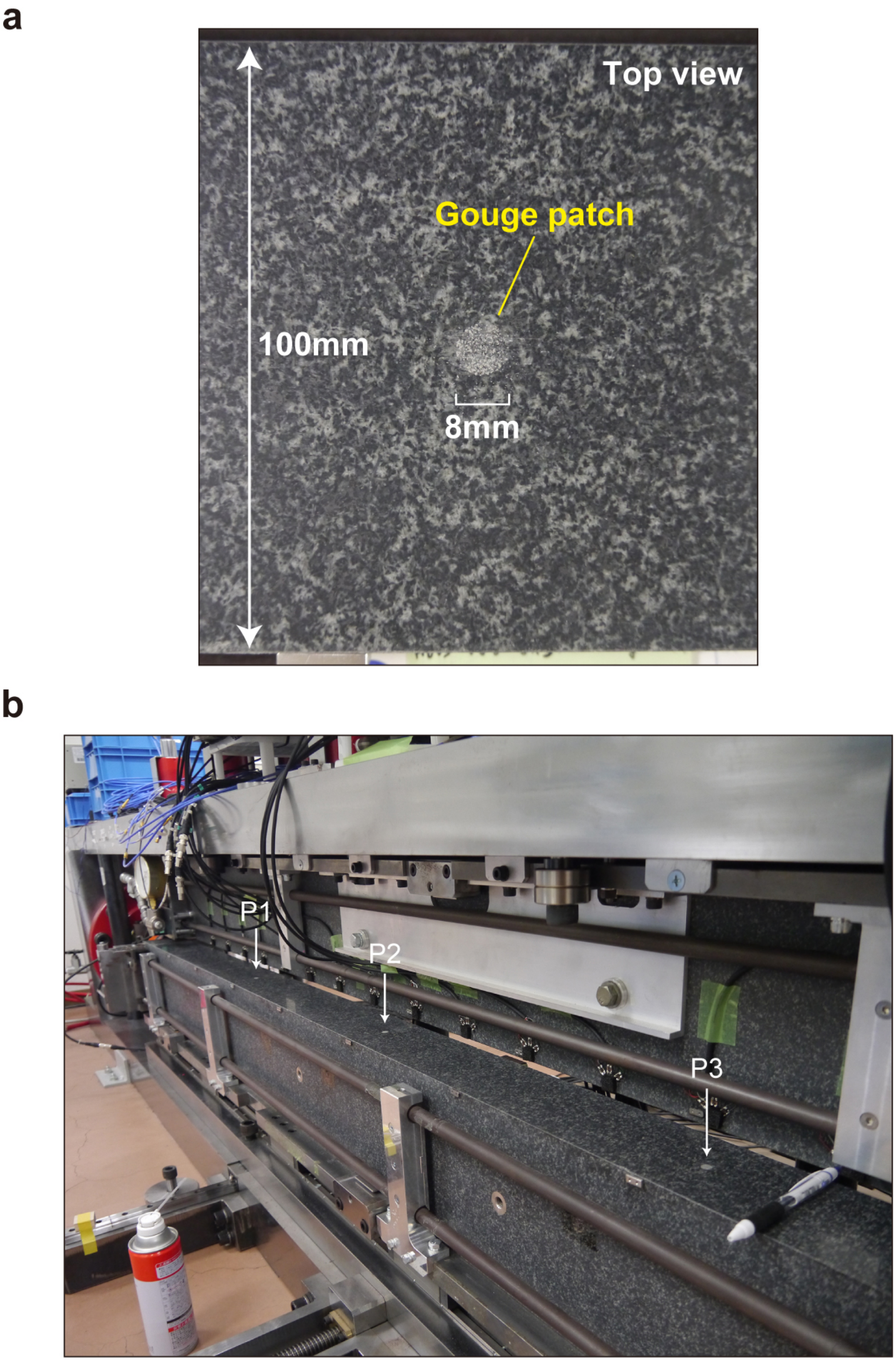

Fig. S3 Photographs of the gouge patch. a, Top view of the GP placed on the fault surface of the bottom rock specimen at patch P3, taken prior to the stick-slip experiment. **b**, Oblique view of the GPs set on the fault. The bottom rock specimen can be pulled back to facilitate the placement of the GPs. The photograph was taken after the removal of the AE sensors, which had been installed on the side surface of the bottom rock specimen.

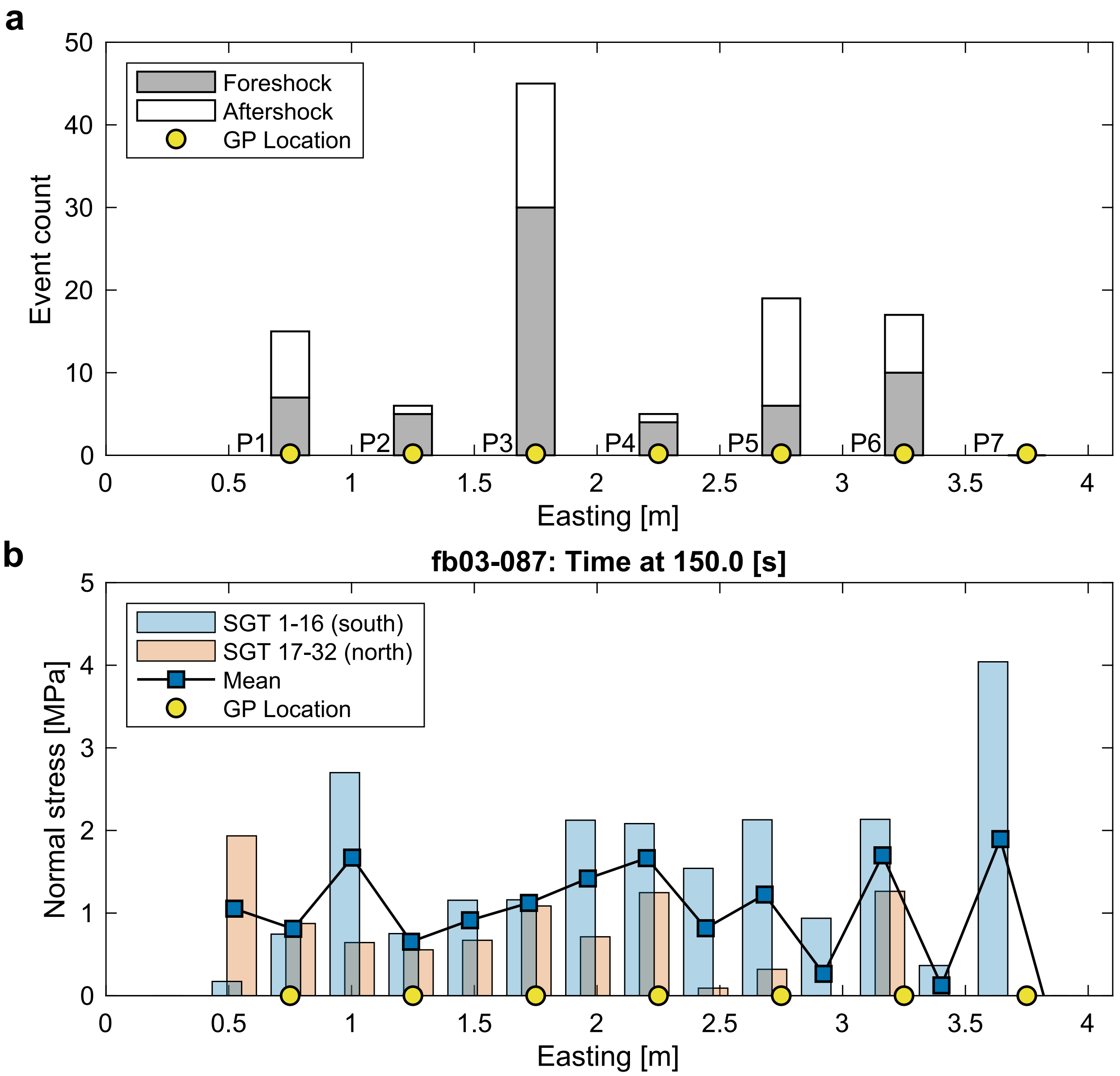

Fig. S4 GP event activity. a, Number of foreshocks and aftershocks observed during the stick-slip experiment. Patch P3 showed the highest level of activity and was used for the main analysis. **b**, Local normal stresses on the fault measured by strain gauges installed on the side surfaces of the top rock specimen. The stress asymmetry between the north and south sides is likely due to a slight contact imbalance along the fault.

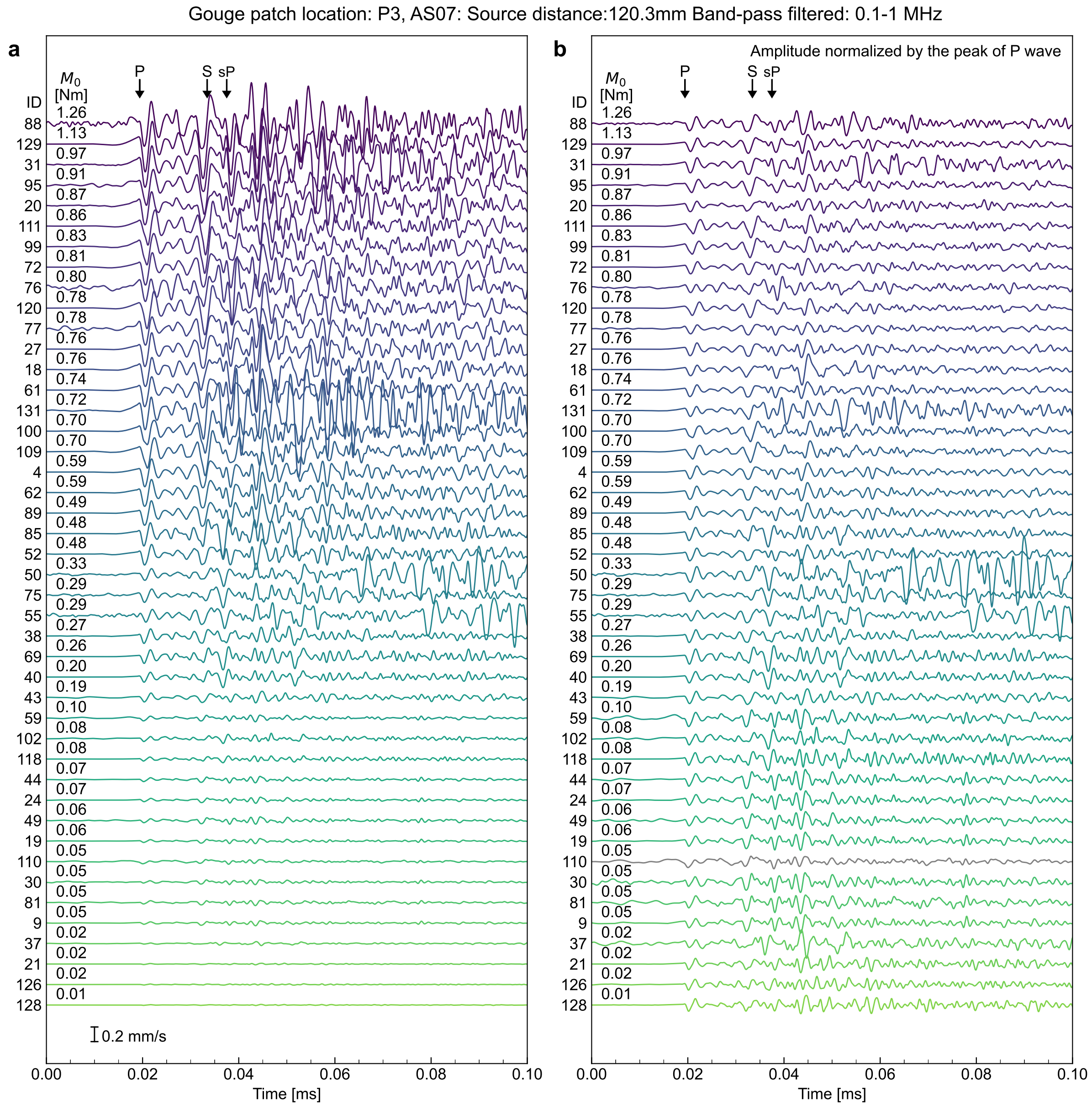

Fig. S5 Collection of AE waveforms. a, c, e, g, Velocity waveforms for fore- and aftershocks generated by P3, recorded by AE sensors AS07, AS08, AS22, and AS23, respectively. **b, d, f, h**, Corresponding waveforms normalized by the P-wave amplitude. Annotations follow the same format as in Fig. 1d of the main text. A total of 44 GP events generated by P3 are displayed. The seismic moment M_0 is computed as the average of values inferred from source time function (STF) fitting across all valid sensors that satisfy the quality thresholds (see Methods). In **b**, **d**, **f**, and **h**, waveforms with low signal-to-noise (S/N) ratios are shown in grey and normalized using a fixed scaling factor prescribed for visualization. In **e** and **f**, the pS-converted wave reflected from the side surface of the rock specimen is also indicated.

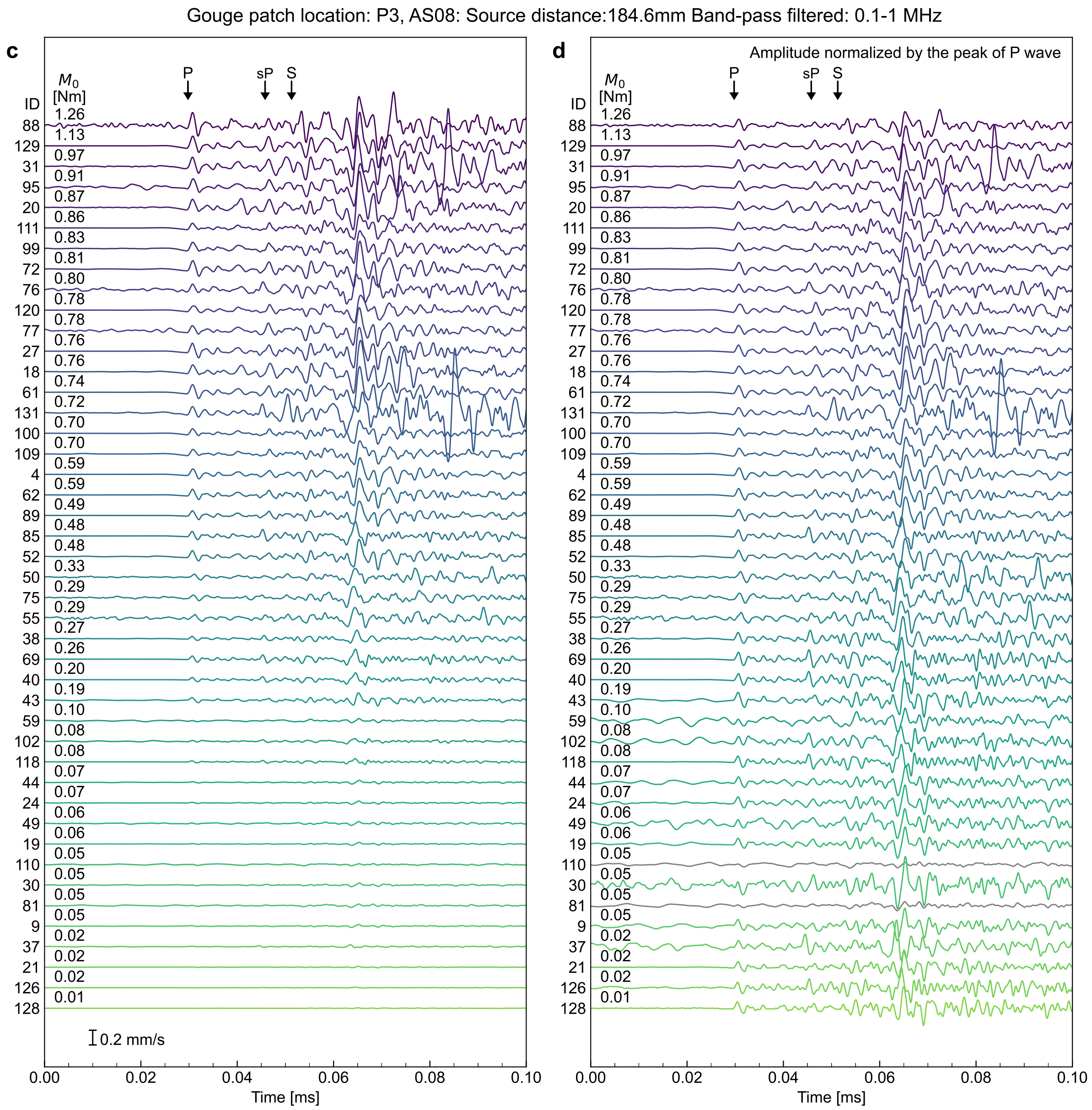

Fig. S5 (continued)

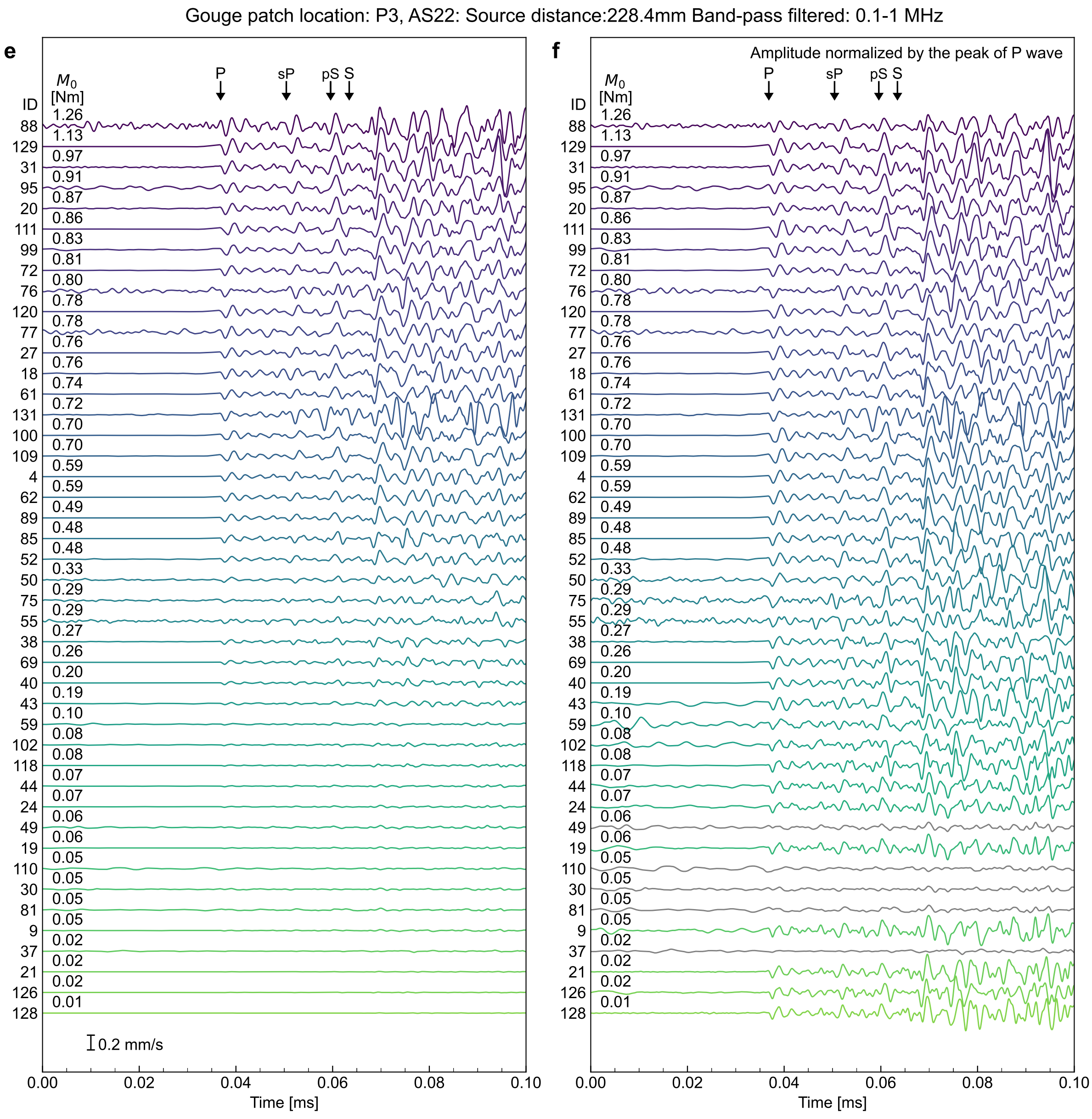

Fig. S5 (continued)

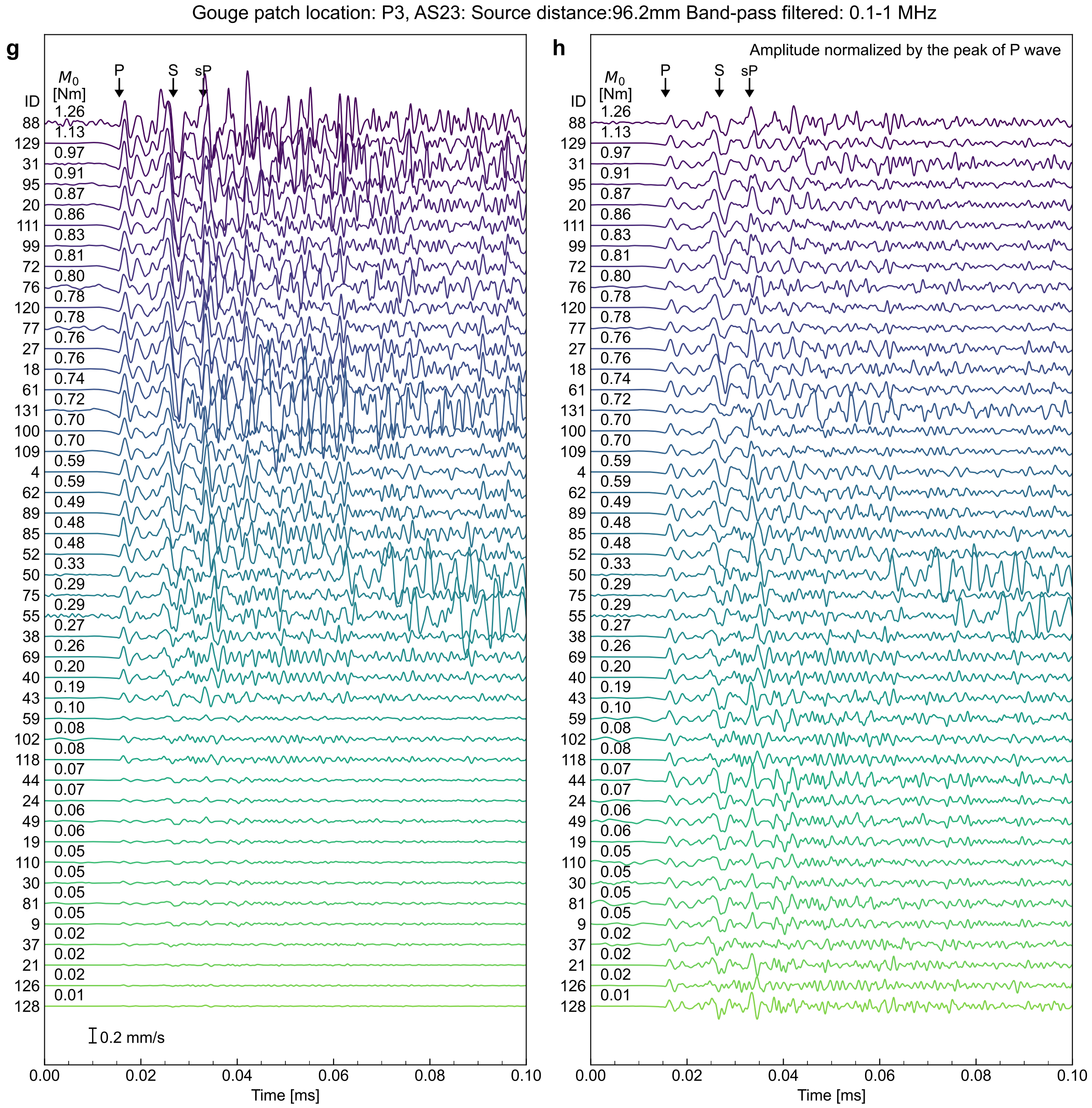

Fig. S5 (continued)

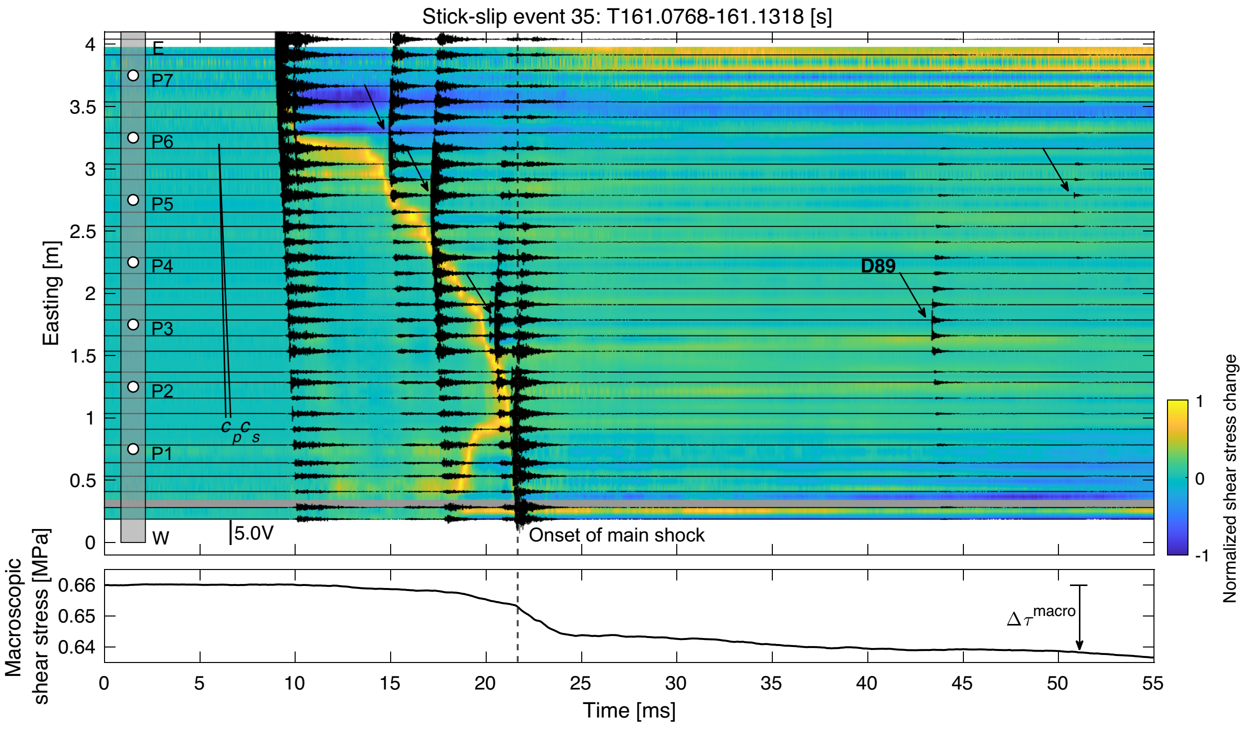

Fig. S6 Observation of GP events during a stick-slip event ID35, including aftershocks, presented in a format similar to Fig. 2a in the main text. Aftershocks were detected during the stick-slip event, with GP event D89 showing a representative aftershock generated by P3.

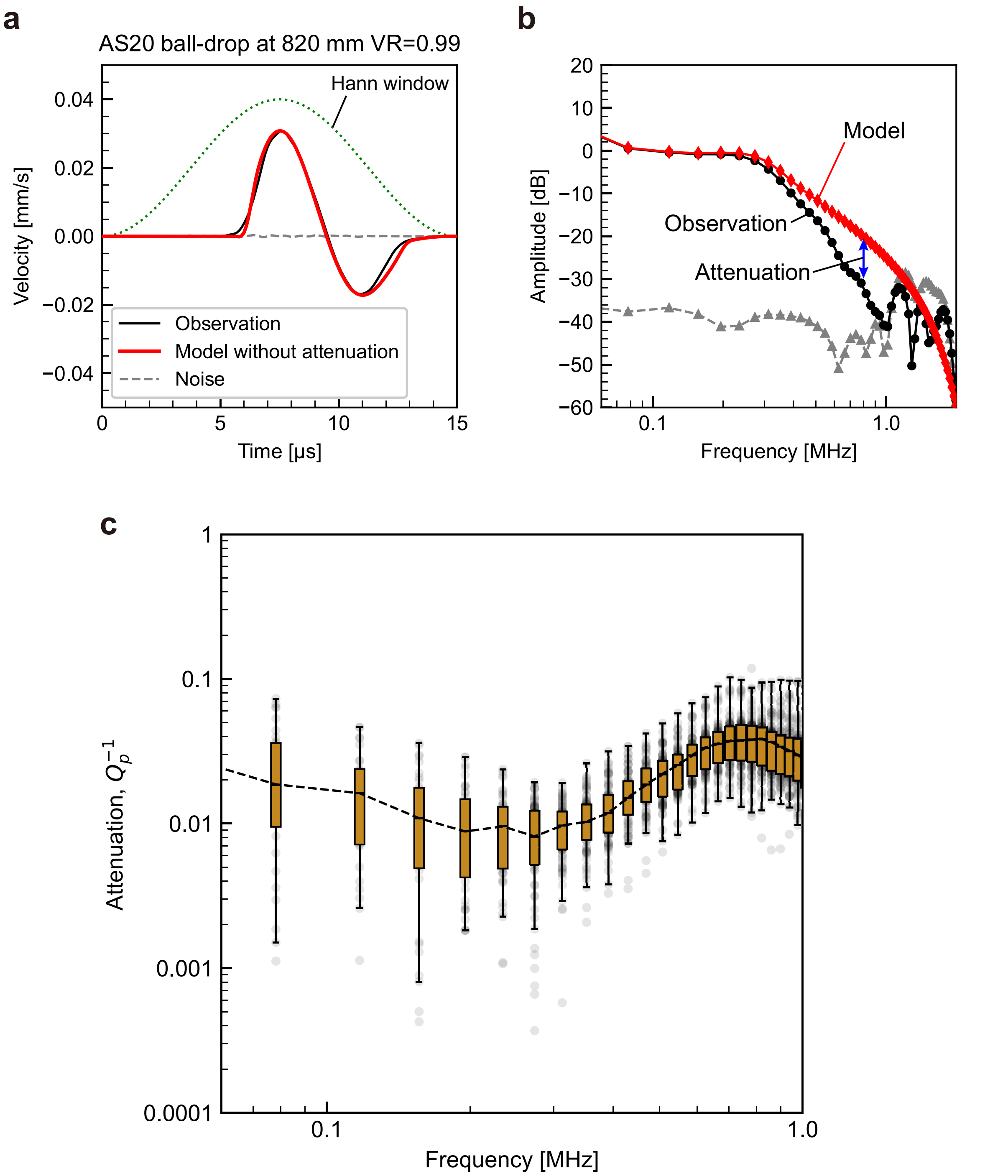

Fig. S7 Evaluation of the frequency-dependent attenuation factor. **a**, Comparison of observed (black) and modeled (red) P-waveforms, with both being windowed using a 15 μs Hann window. The gray dashed line represents the noise level. **b**, Comparison of P-wave spectra from **a**, where the difference between the observed and modeled spectra reflects the attenuation. **c**, Statistics of the attenuation estimates. The box plot displays the first and third quartiles, with individual values shown as grey circles. The dashed line represents the median attenuation model, which is used for attenuation correction in the source parameter estimation.

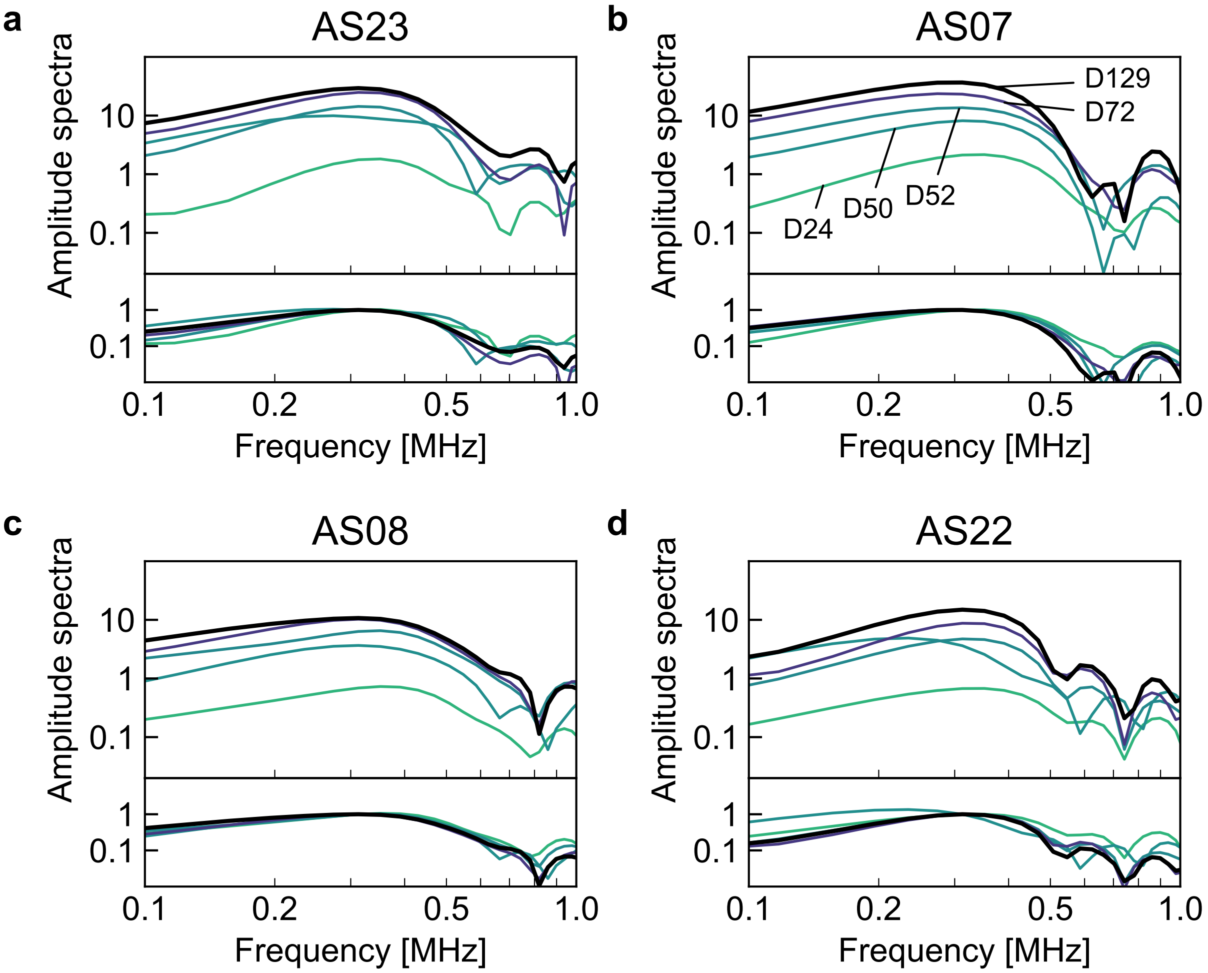

Fig. S8 Spectral ratio analysis. a-d, Amplitude spectra of the P-wave windows for the four AE sensors located near P3. The thick black line represents the spectra for the largest event (D129), while the other lines show spectra for representative non-self-similar GP events (D24, D50, D52, and D72), with the line color corresponding to the seismic moment (M_0). The bottom panel of each subplot shows the amplitude spectra normalized at 0.3 MHz.

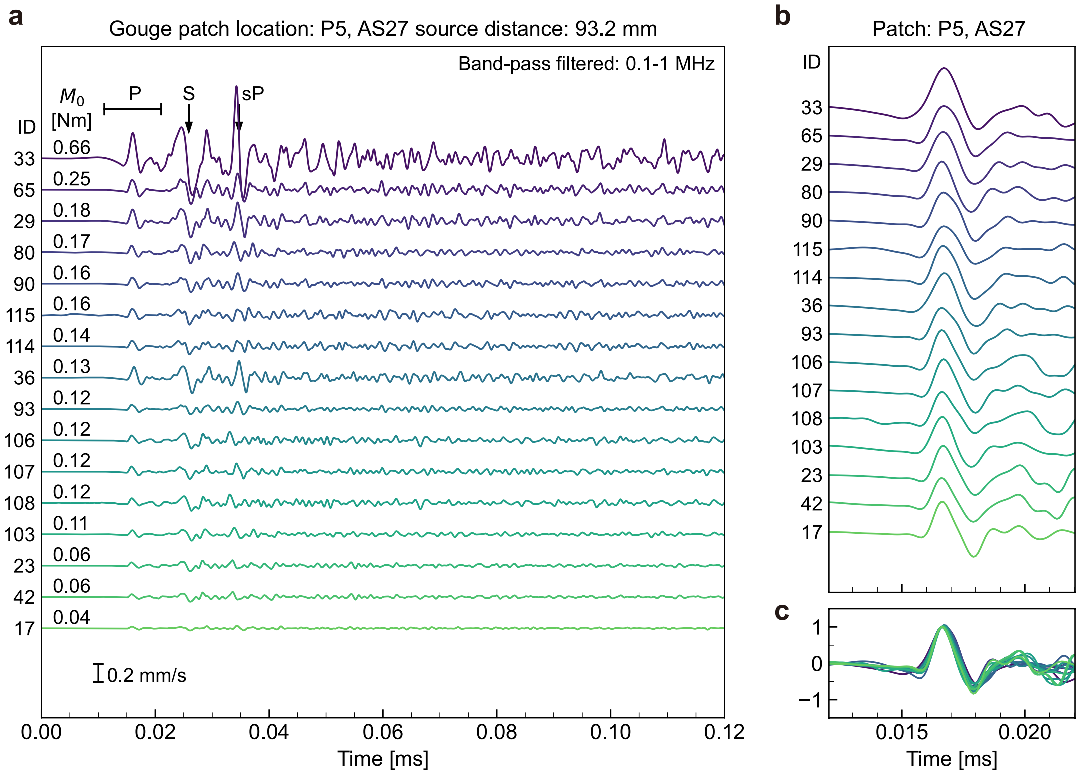

Fig. S9 Observed non-self-similarity at patch P5. **a**, AE waveforms recorded at AS27 for 16 events generated on gouge patch P5. Seismic moments were estimated through preliminary waveform fitting using synthesized Green's functions. The same preprocessing as in Fig. 1c of the main text was applied to these waveforms. **b**, Normalized P-waveforms. **c**, Superposition of normalized P-waveforms, highlighting the consistency in source duration across events of different magnitudes.

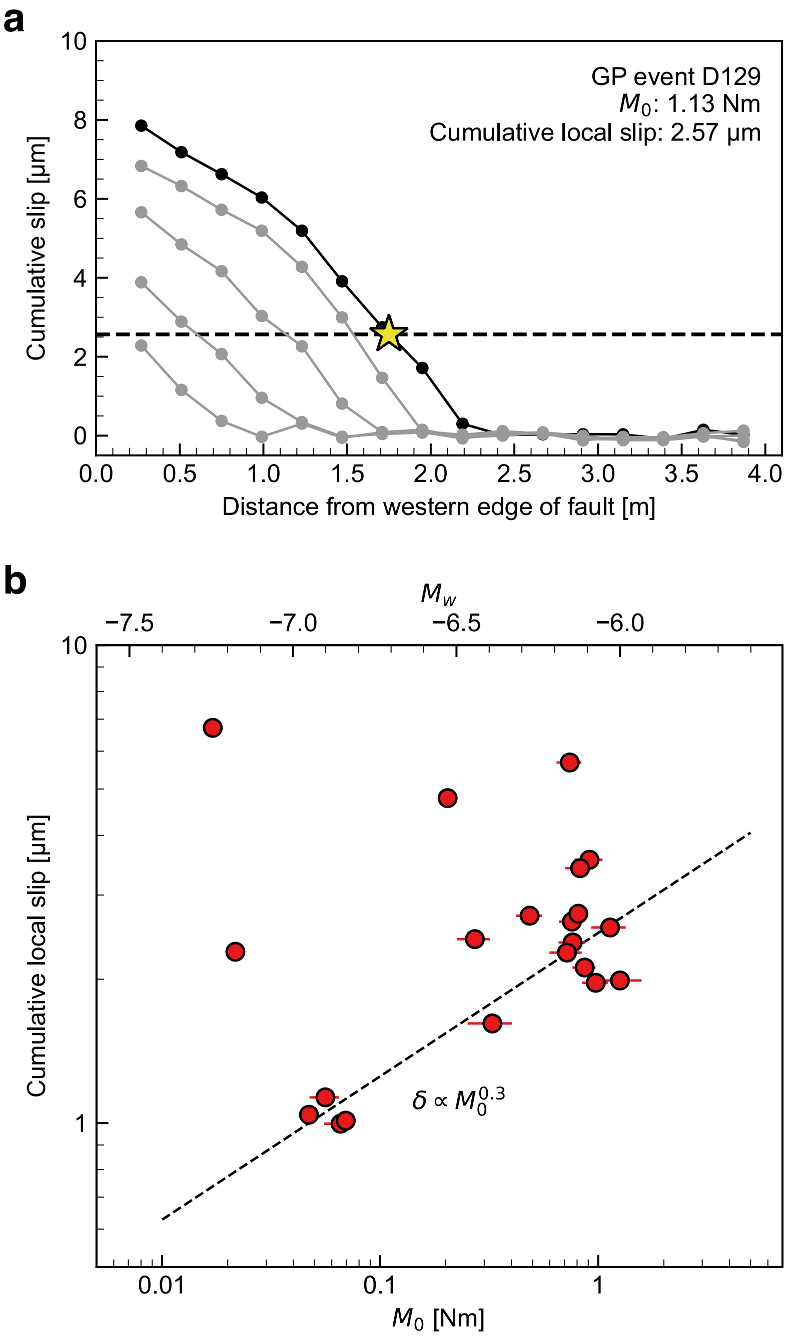

Fig. S10 Correlation between seismic moment and cumulative local slip. a, Evaluation of cumulative local slip during a GP-generated foreshock. Grey lines indicate the evolution of preslip, while the black line represents the slip profile at the onset of the gouge event. The cumulative local slip was obtained by linear interpolation in time and space using 16 gap sensors at the GP (indicated by the star), which is shown by the horizontal dashed line. **b**, Comparison of seismic moment with cumulative local slip. A total of 21 foreshocks were selected using thresholds similar to those described in the “Fitting STF” subsection of the Methods. Markers and error bars denote the mean and standard error of the seismic moment, respectively. The dashed line indicates a reference slope with a scaling exponent of 0.3.

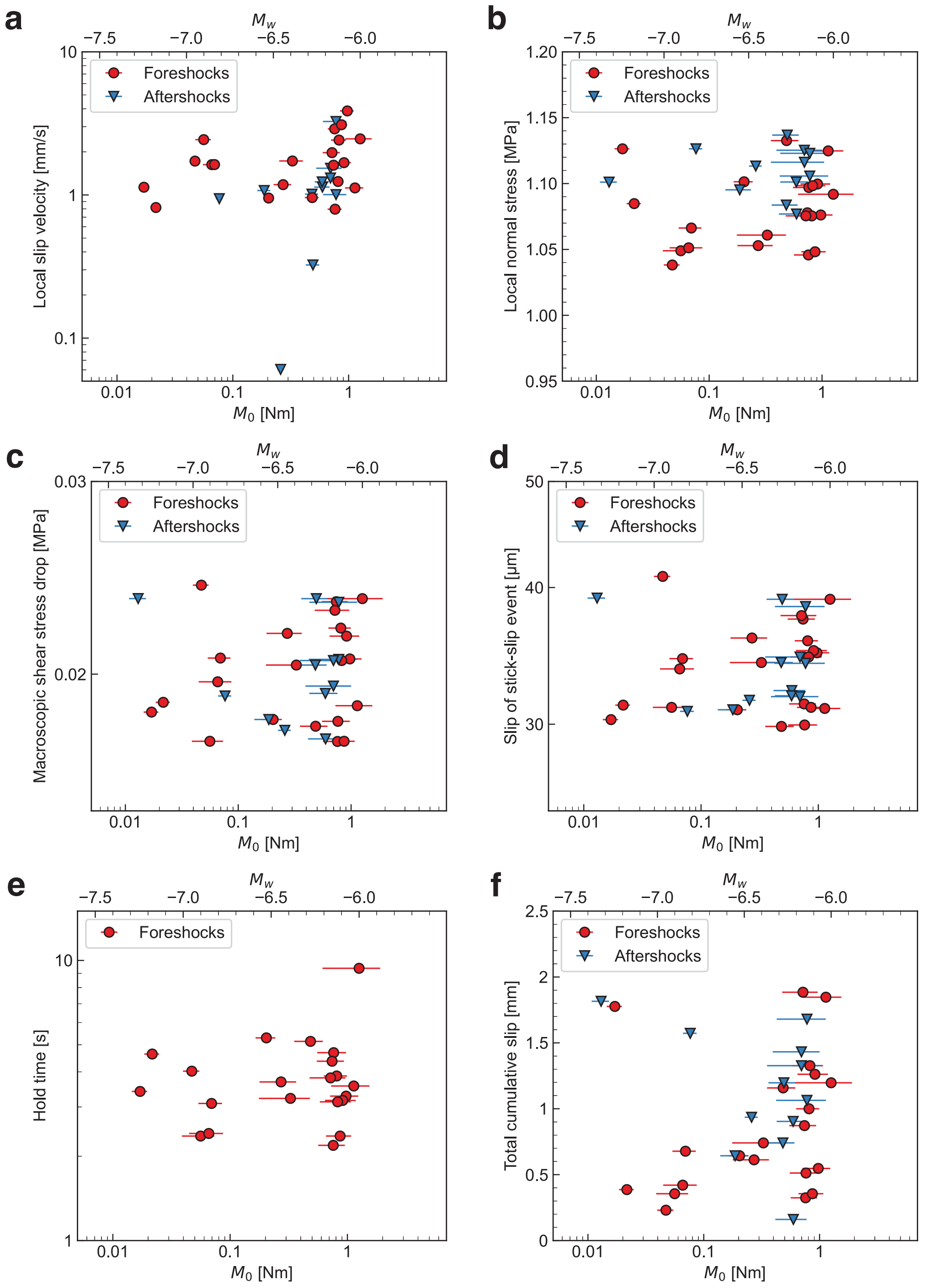

Fig. S11 Relationships between the seismic moment of GP events and various recorded physical parameters. **a**, Local slip velocity. Circles and triangles denote foreshocks and aftershocks, respectively. Horizontal error bars represent the standard error of seismic moment estimated from the four AE sensors near the P3. **b**, Local normal stress. **c**, Macroscopic shear stress drop. **d**, Coseismic slip of the associated stick–slip event. **e**, Hold time (evaluated only for foreshocks for clarity). **f**, Total slip accumulated over successive stick–slip events.

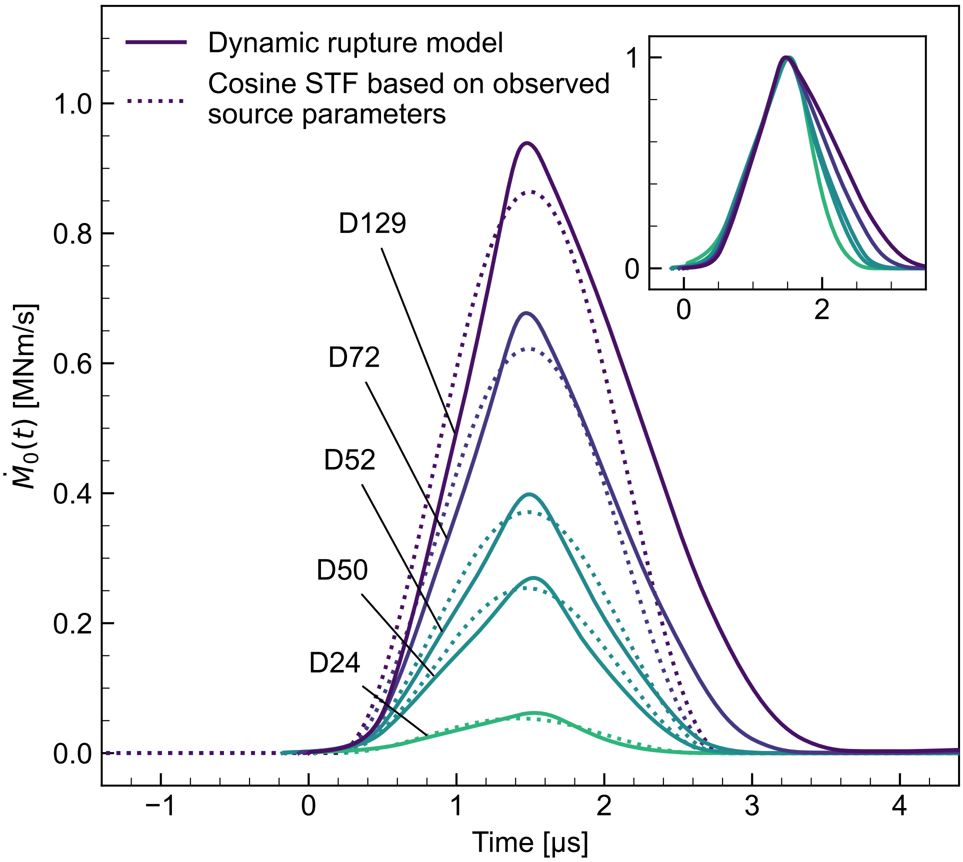

Fig. S12 Dynamic rupture modeling using slip-weakening law without self-healing. The initial stress and frictional conditions are identical to those in Figs. 4a–c (Table S2) of the main text, except that frictional self-healing is not included. The normalized STFs shown in the upper-right panel reveal deviations from the non-self-similar scaling.

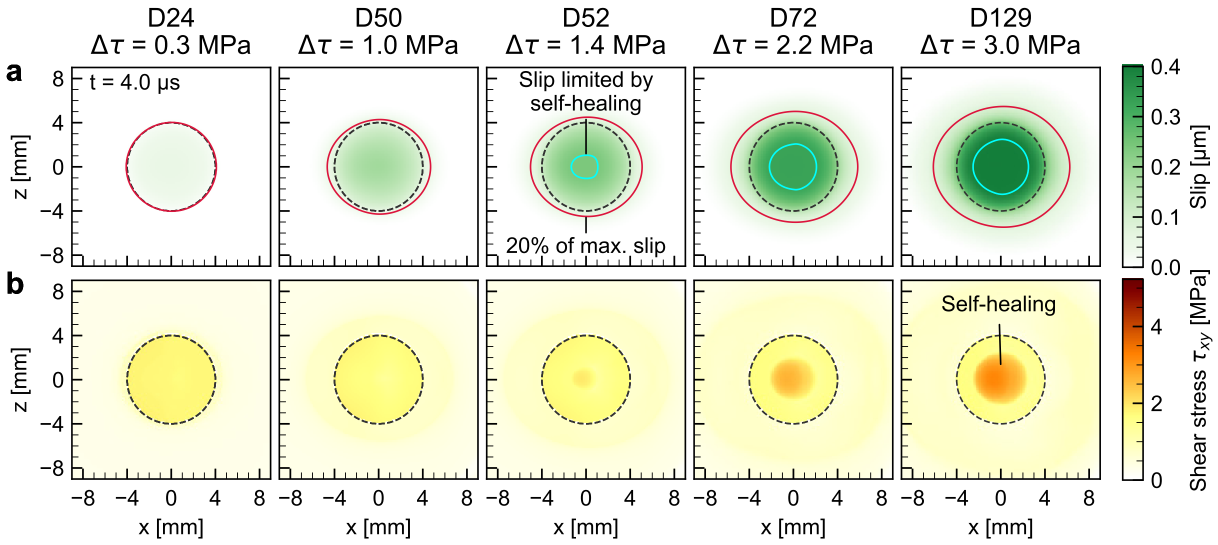

Fig. S13 Comparison of slip and shear stress change for the target events. Each panel shows the final snapshot (t = 4.0 μs) of the dynamic rupture simulations with self-healing friction. Δτ denotes the prescribed dynamic stress drop within the gouge patch. In the present framework, seismic moment M_0 scales with stress drop; accordingly, the spatial extent of the region in which self-healing is activated increases with M_0.

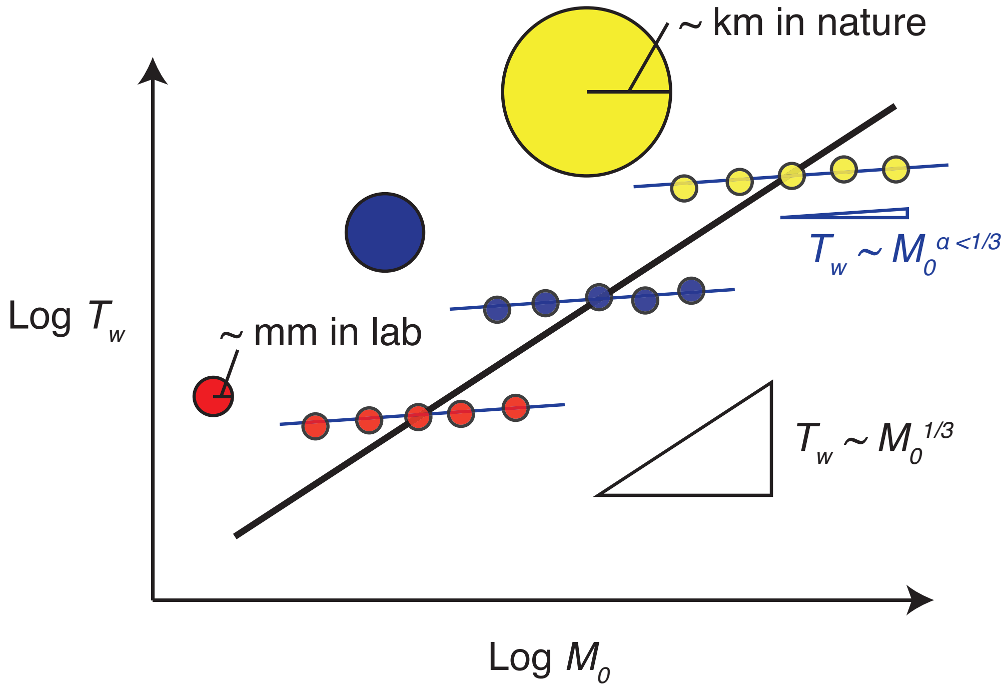

Fig. S14 Schematic illustration of potential scaling behavior for different patch sizes. The thick black line represents the self-similar scaling $T_w \sim M_0^{1/3}$ for different patch sizes. Thin blue lines indicate non-self-similar scaling branches controlled by the patch size.

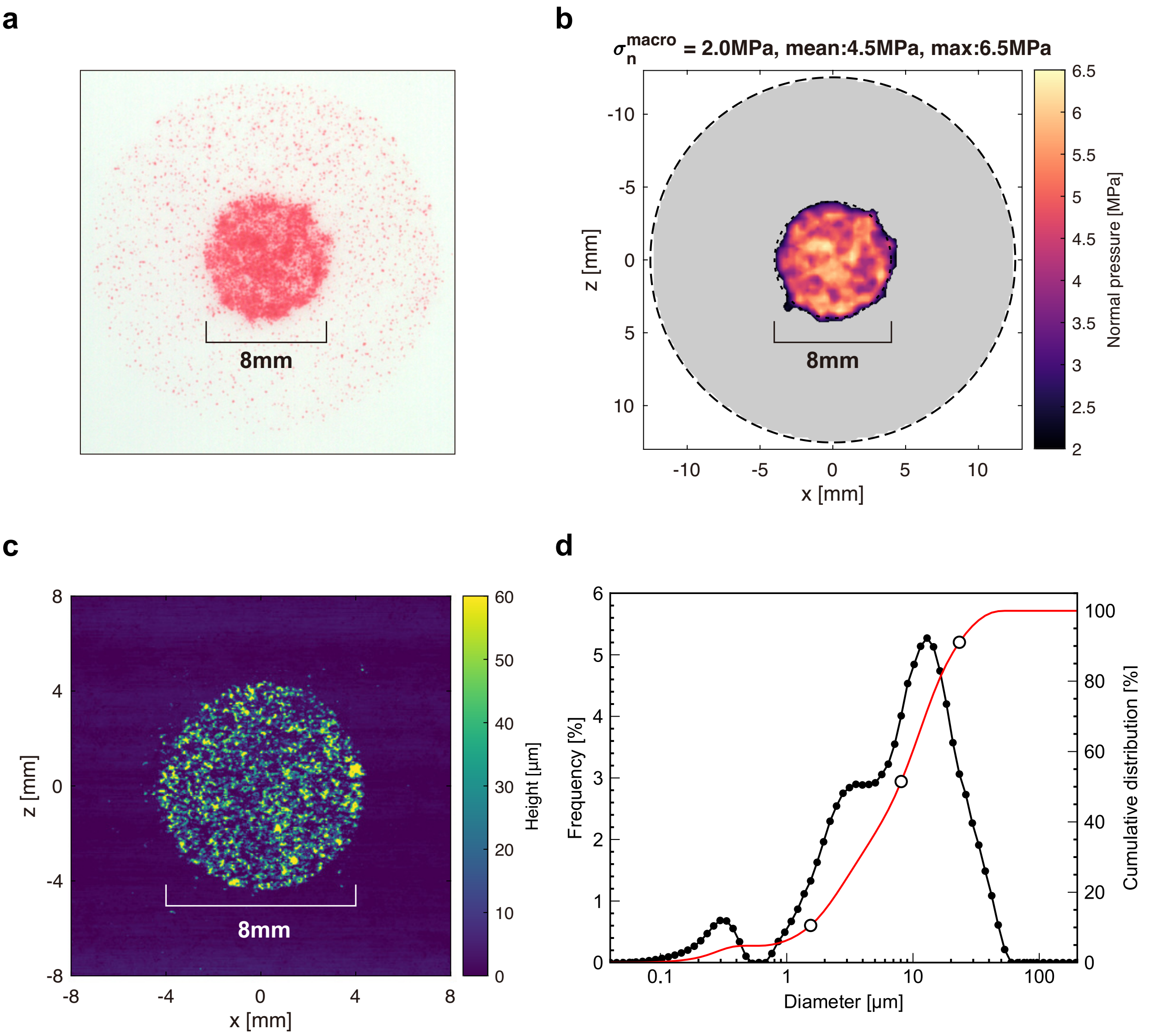

Fig. S15 Measurements of GP physical properties. a, Pressure-sensitive film discolored by normal pressure of 2 MPa. The black-and-white contrast was adjusted for visualization purposes. To quantify the pressure from the color density, a color calibration base was required and used according to the instructions provided by `Prescale Mobile`. **b**, Normal pressure distribution. The increased pressure on the GP is attributed to the presence of a topographic bump. Discoloration was also observed in the outer area of the base rock specimen (highlighted in grey), indicating direct contact with the bare rock surface. The measured values were near the lower detection limit, suggesting a pressure level comparable to the macroscopic normal stress (2 MPa). **c**, Topography of the GP. Note that a different GP from **a** and **b** was used for the height measurement. **d**, Particle size distribution of the gouge. The black and red lines show the interpolated frequency distribution of particle diameters and the cumulative distribution, respectively. The percentile diameters are $D_{10} = 1.4$ μm, $D_{50} = 8.2$ μm, and $D_{90} = 24.0$ μm, as indicated by the open circles.

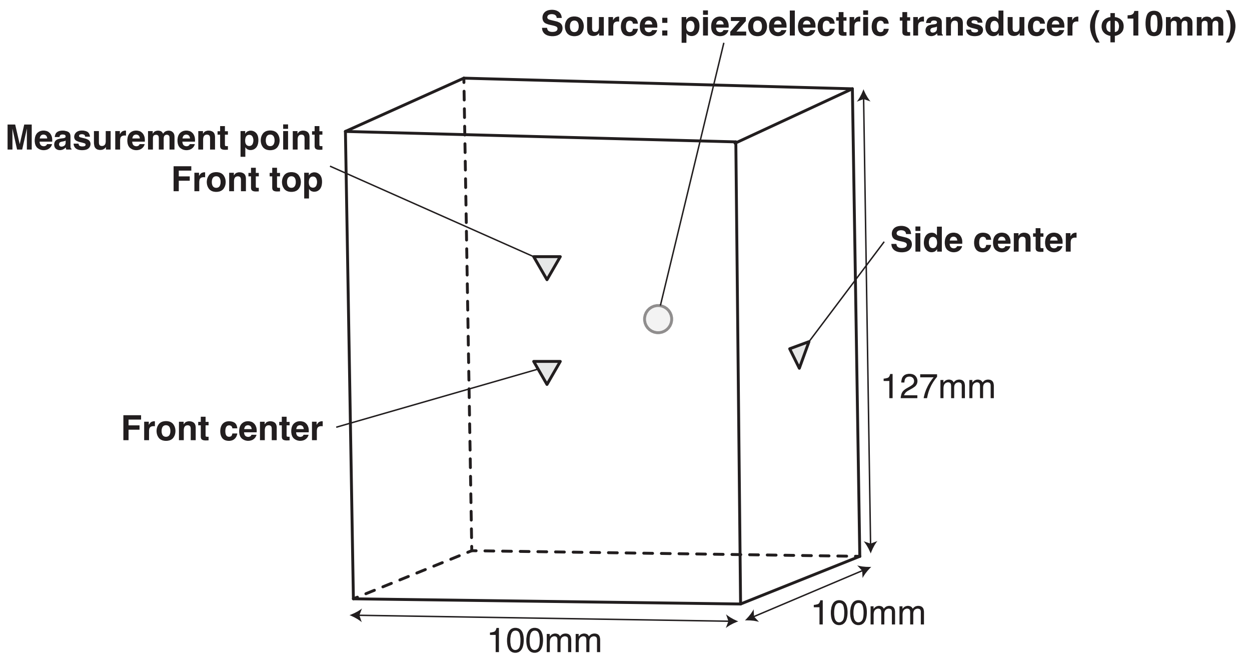

Fig. S16 Schematic of AE sensor calibration using a piezoelectric transducer (PZT) on a steel block. To enhance the signal-to-noise ratio (S/N) while avoiding sensor overload, the PZT was excited at 200 V for LDV measurements and 100 V for the AE sensor. The linearity of the source with respect to the input voltage was verified using LDV, showing that the 200 V waveform scales by a factor of 1.79 to match that at 100 V. We sequentially recorded waveforms at three different locations to validate the robustness of the sensor response calibration.

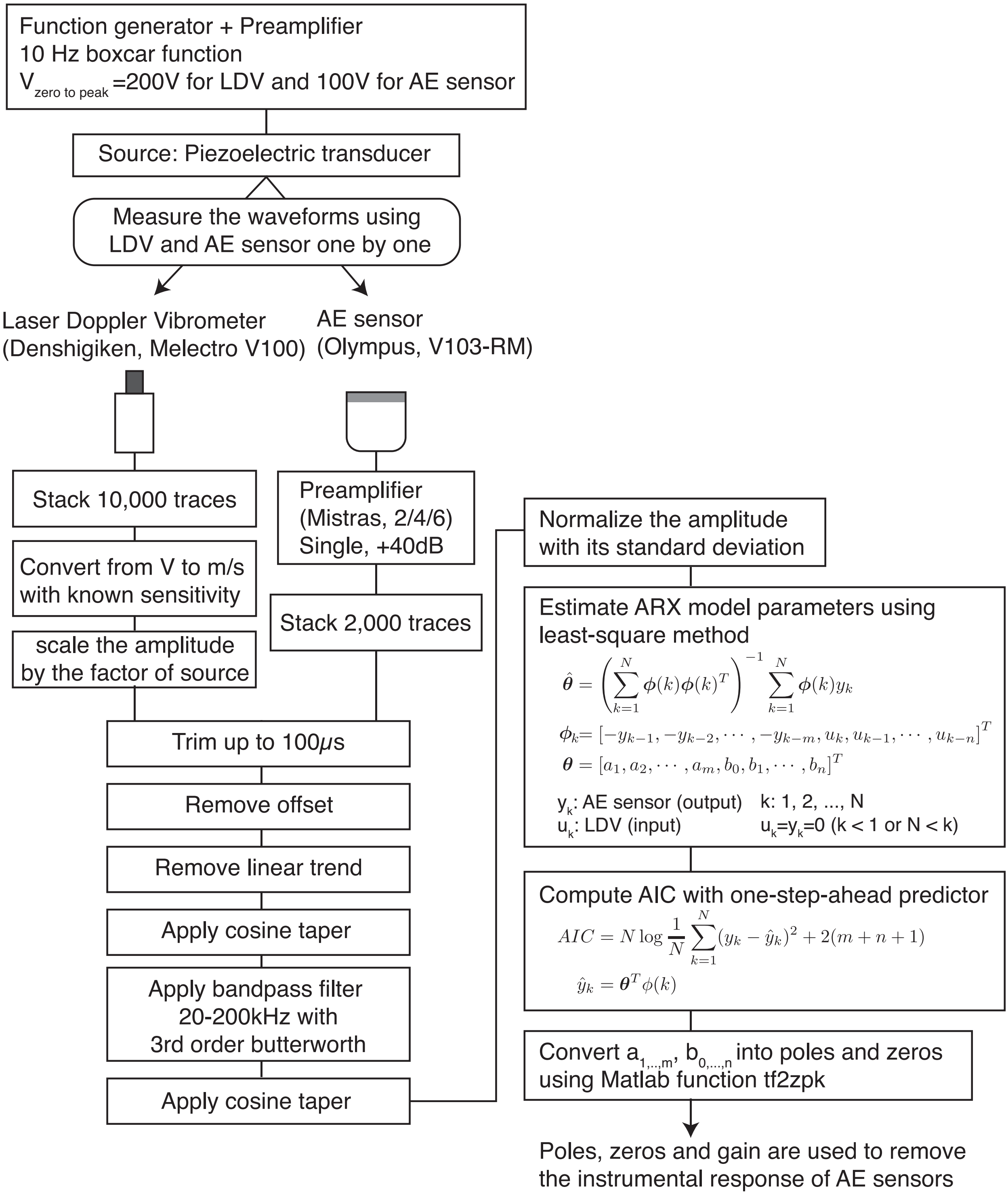

Fig. S17 Process flow for calibrating AE sensor response.

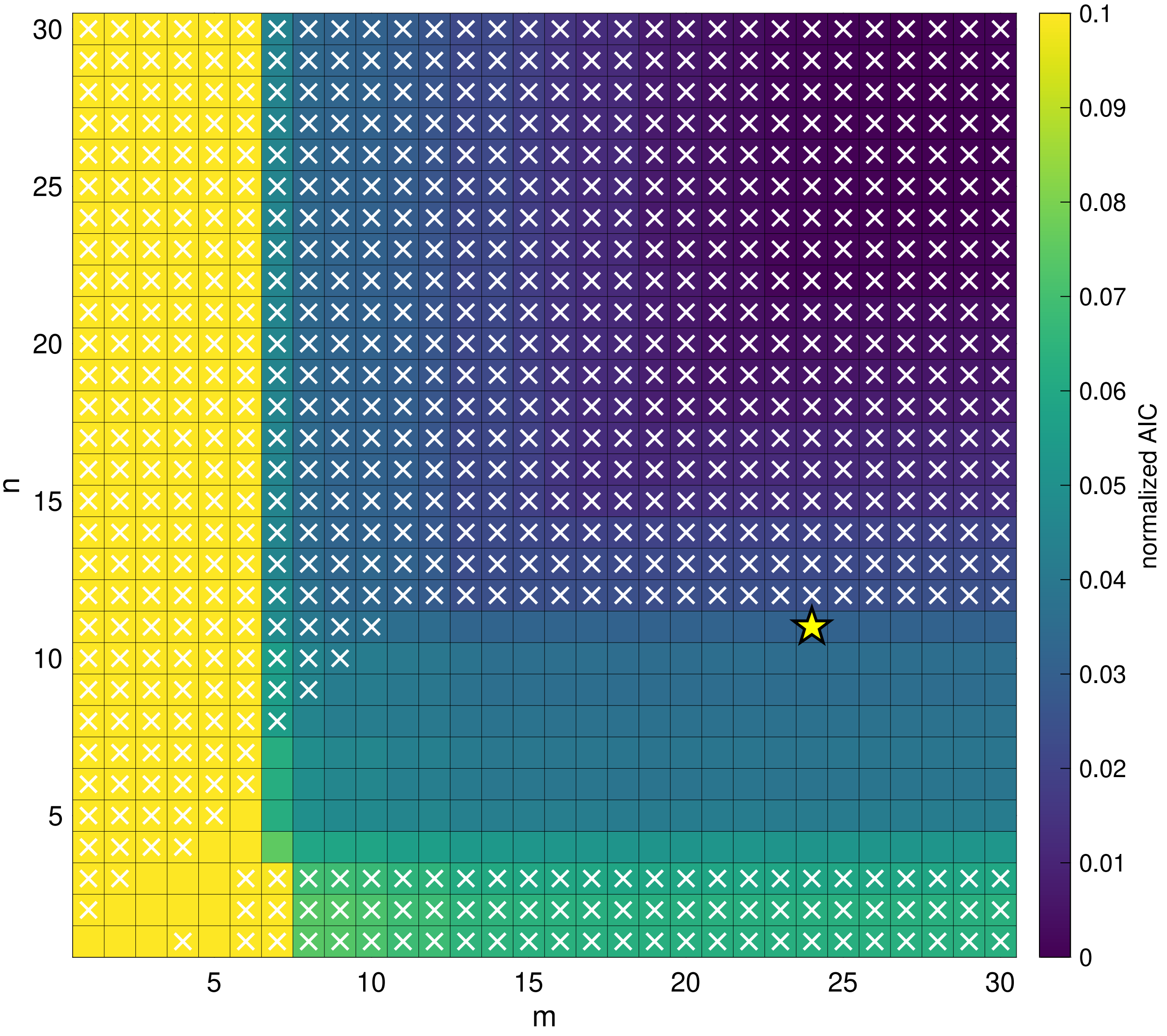

Fig. S18 Case study of the Akaike Information Criterion (AIC) as a function of the number of poles (m) and zeros (n). The color map shows the normalized AIC values, scaled between their minimum and maximum. Cross markers indicate invalid (m, n) combinations where the least-squares estimation was unstable or where the estimated poles and zeros were located outside the unit circle. Improper configurations, such as those with $n > m$, are masked. The yellow star indicates the selected model corresponding to the minimum AIC value.

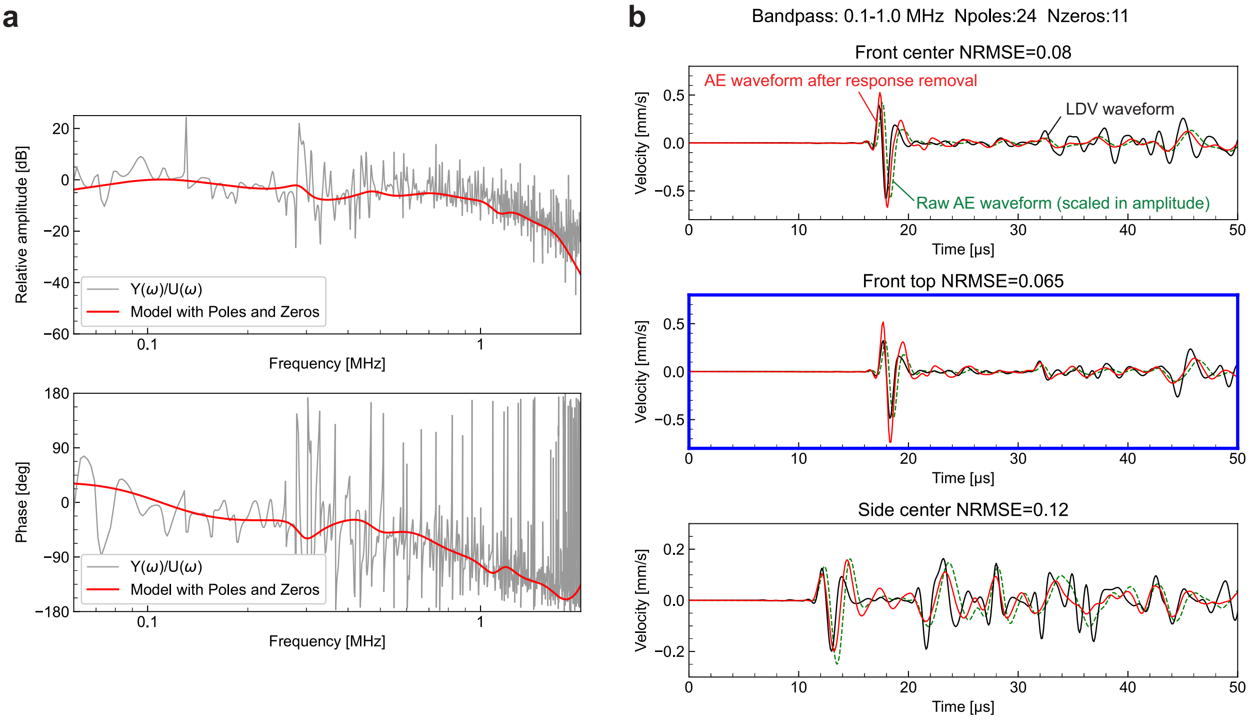

Fig. S19 Calibration of AE sensor response. a, Bode plot of the AE sensor. The grey line represents the sensor response, estimated as $Y(\omega)/U(\omega)$, where the Fourier spectrum of the AE sensor is divided by that of the LDV. The red line corresponds to the ARX model with 24 poles and 11 zeros. **b**, Evaluation of response correction performance at three measurement locations. Black, green, and red lines correspond to the LDV measurement, AE sensor output, and the AE waveform after response correction, respectively. The middle panel (blue box) shows the case of recording at the front-top of the block (Fig. S16) used for estimating the poles and zeros. The top and bottom panels show results at two additional measurement locations. Waveform matching between the LDV and corrected AE signals is assessed using the normalized root mean square error (NRMSE).

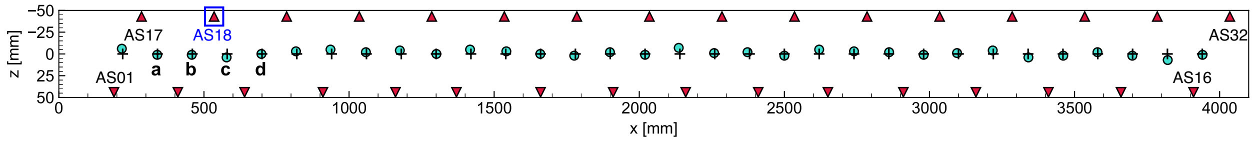

Fig. S20 Locations of ball-drop impacts on the fault. The cross markers indicate the prescribed target locations, while the cyan circles represent the relocated impact positions. Triangles mark the locations of AE sensors. For visualization purpose, the fault's aspect ratio is scaled by a factor of three in the z-direction. The labels (a–d) and the highlighted sensor (AS18) correspond to the ball-drop sources and the receiver used for the waveform examples shown in Fig. S23.

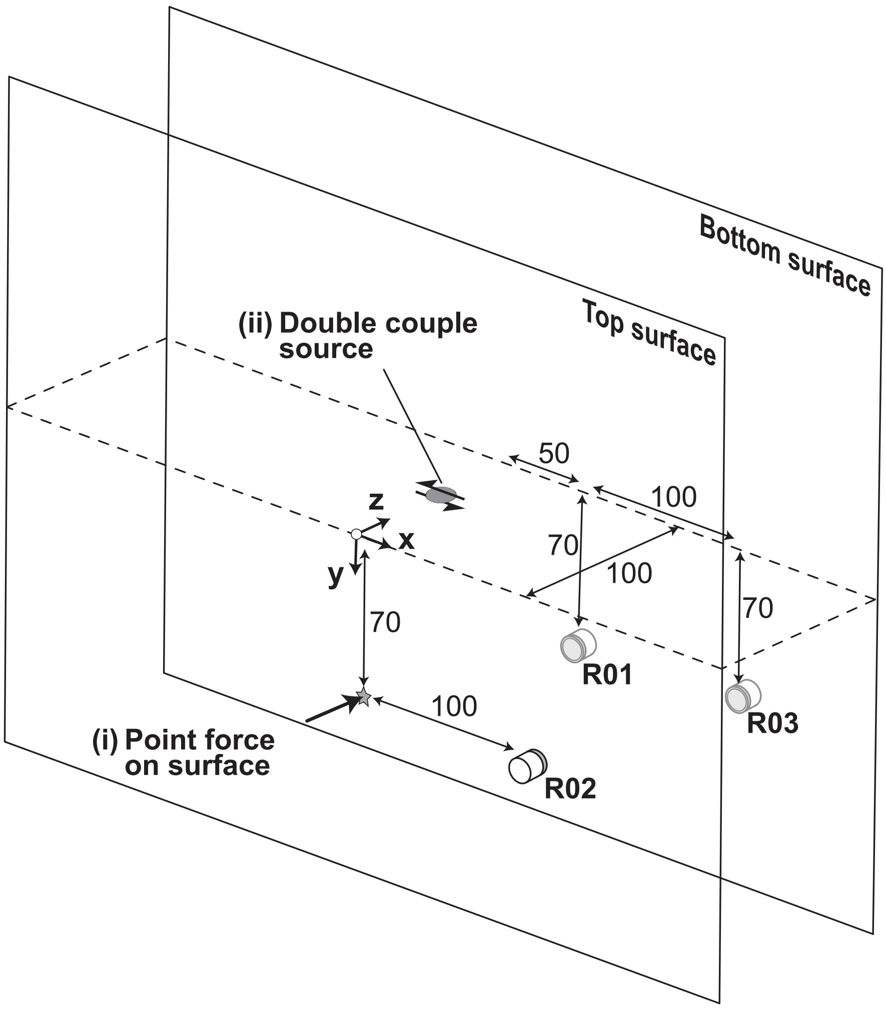

Fig. S21 Model setup of sources and sensors for the cross-verification of the extended `OpenSWPC`. Three virtual sensors were placed on the top and bottom surfaces, and wave propagation was simulated for two scenarios: (i) a point force applied to the side surface and (ii) a double-couple source representing a seismic event on a fault. The computational domain was modeled as an infinite plate with a uniform velocity structure, omitting the explicit fault geometry. The elastic constants matched those of the rock specimen used in the main analysis. Green's functions between the sources and sensors were calculated and then convolved with a synthetic cosine force-time function for case (i) and a moment-rate function for case (ii) to generate synthetic waveforms for comparison.

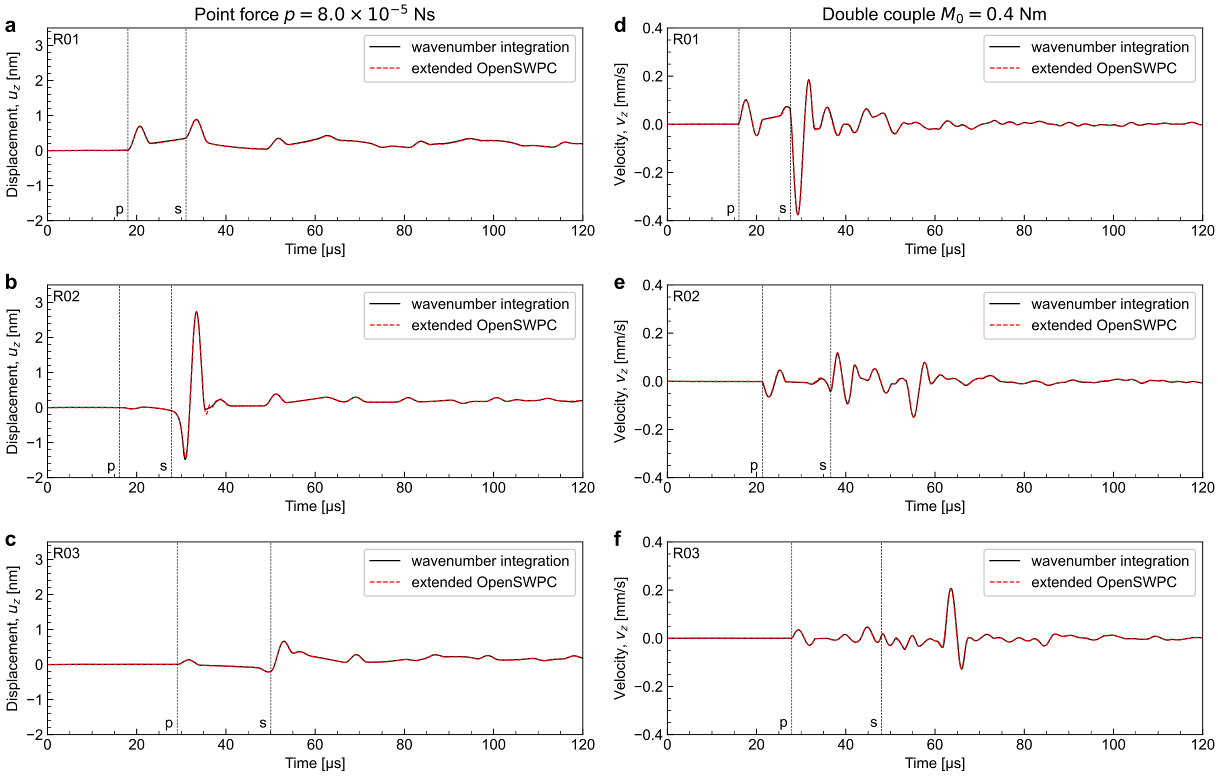

Fig. S22 Cross-verification of the modeled waveforms. a–c, Comparison of waveforms generated by a point force. Black and red lines represent waveforms computed using the reference program `Computer Programs in Seismology` (Herrmann, 2013) and the extended `OpenSWPC`, respectively. The point-force source used a cosine function with a momentum $p = 8.0 \times 10^{-5}$ Ns and a source duration of 5.0 μs. Dashed vertical lines indicate the P- and S-wave arrival times. No filtering was applied. **d–f**, Comparison of waveforms generated by a double-couple source with a seismic moment $M_0 = 0.4$ Nm and the same source duration. The extended `OpenSWPC` accurately reproduces the reference waveforms in both source configurations.

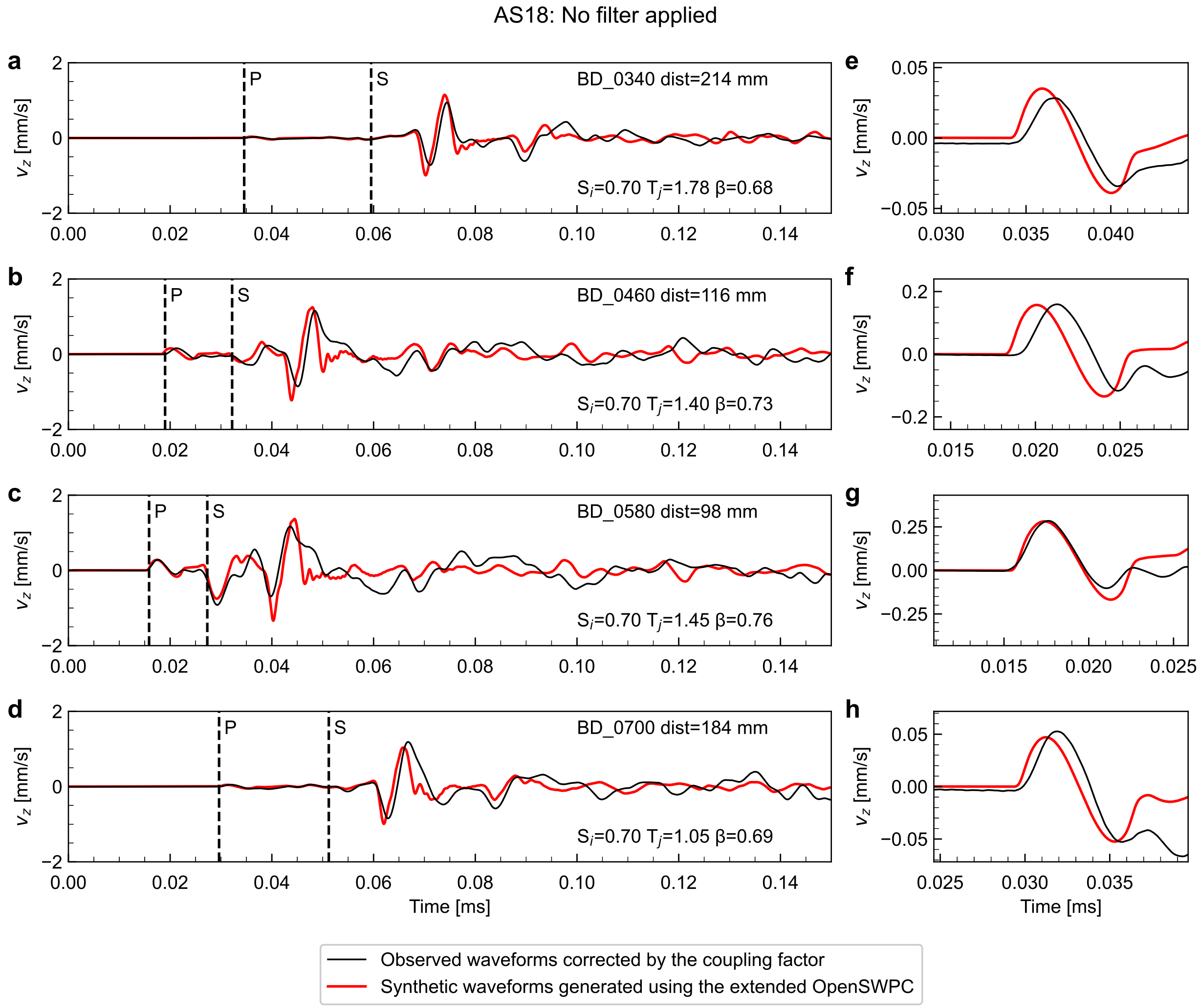

Fig. S23 Comparison of observed (black) and modeled (red) velocity waveforms from ball-drop impacts used for sensor coupling calibration. a-d, Waveforms generated by ball-drop impacts at different source locations and recorded by AS18, as shown in Fig. S20. The observed waveforms were shown after correction for sensor coupling, ball-drop impact characteristics, and aperture effect. Vertical dashed lines indicate the theoretical arrival times of the P- and S-waves. **e-h** magnified views of the P-wave window.

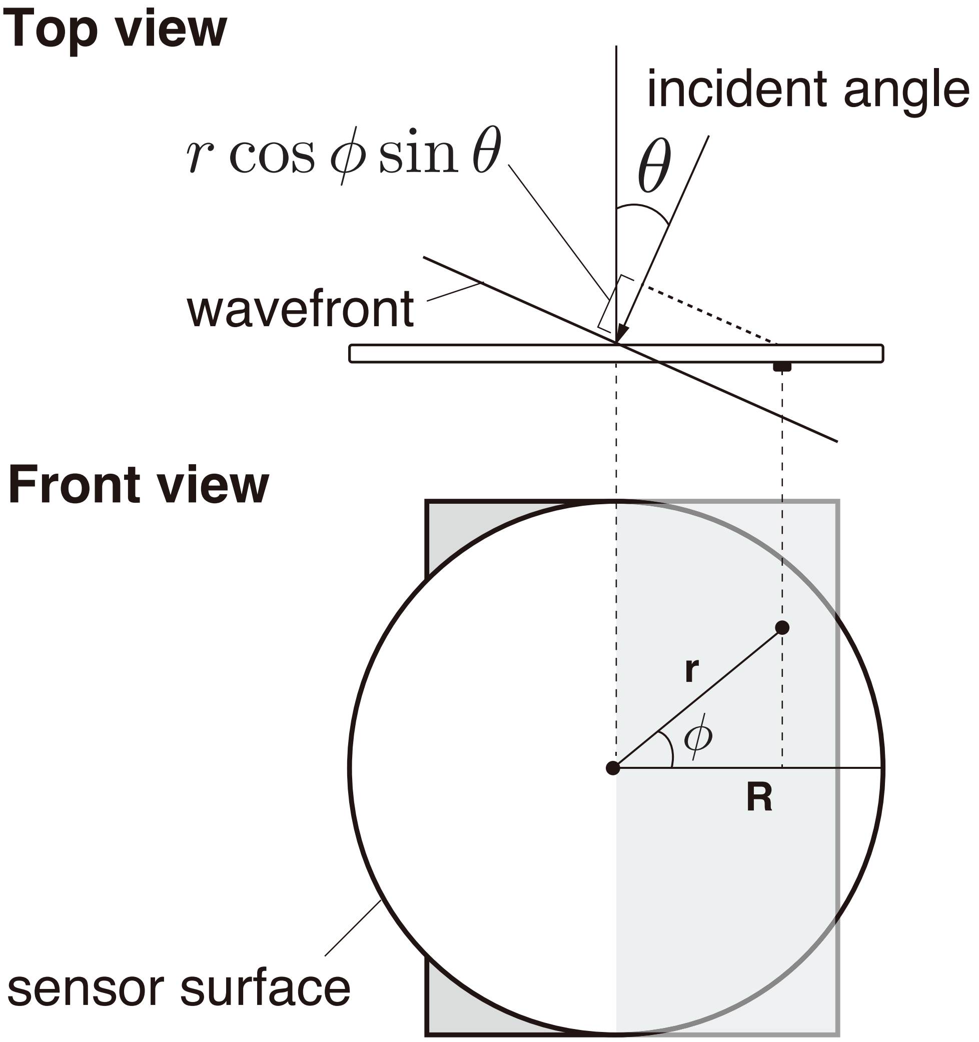

Fig. S24 Schematic showing the geometry of the incident angle of a plane wave propagating toward the AE sensor.

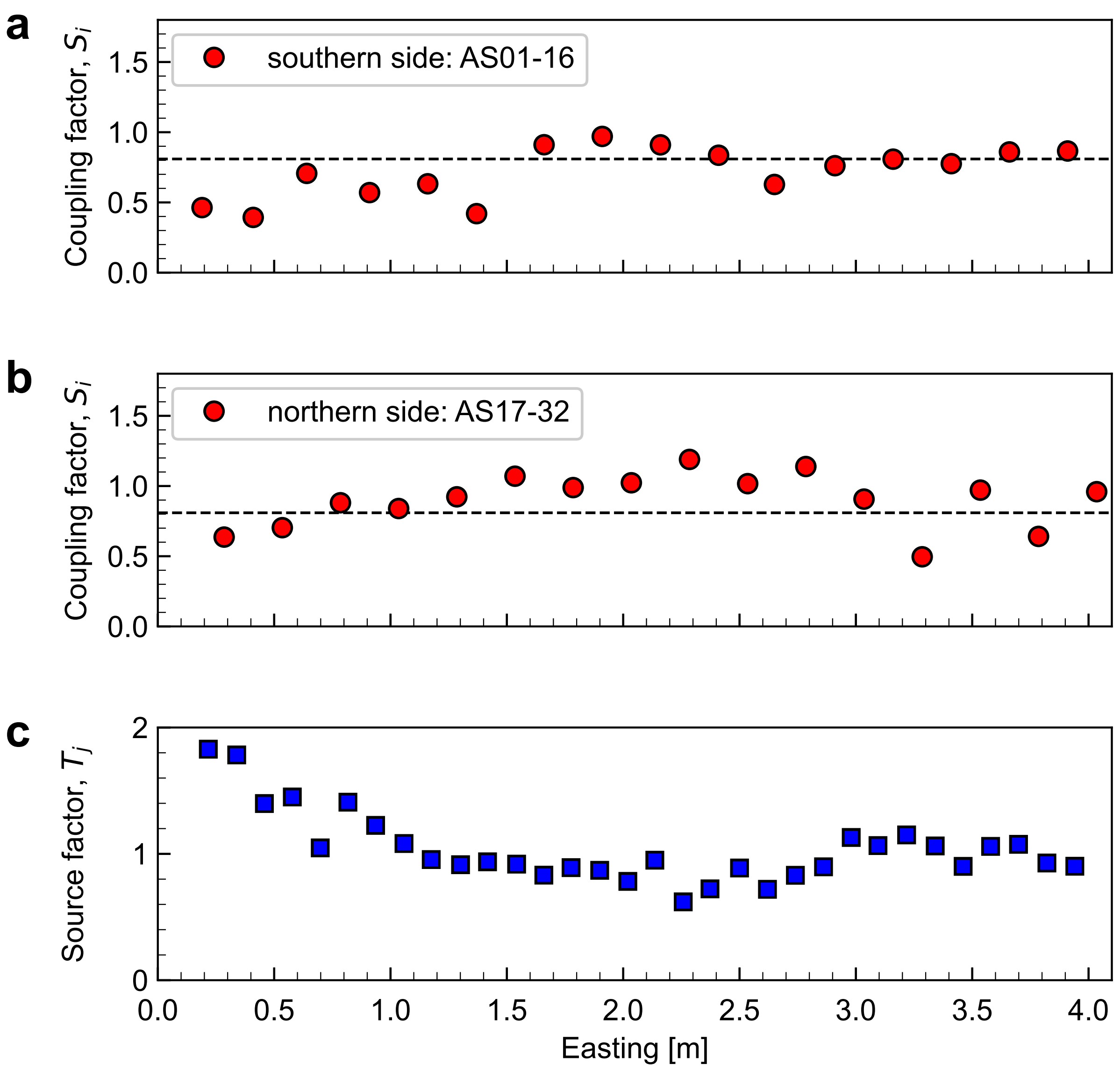

Fig. S25 Result of calibration for the sensor coupling factors. a-b, Optimized sensor coupling factors, S_i, for the AE sensors installed on the southern and northern sides of the rock specimen, respectively. The horizontal dashed line indicates the average coupling factor of 0.81. **c**, Correction factor, T_j, for the ball-drop source impact. The angular frequency, ω, was optimized to 270 kHz. Although this value may be slightly overestimated, it lies within a reasonable range for the dominant frequency of the observed P-wave pulses.

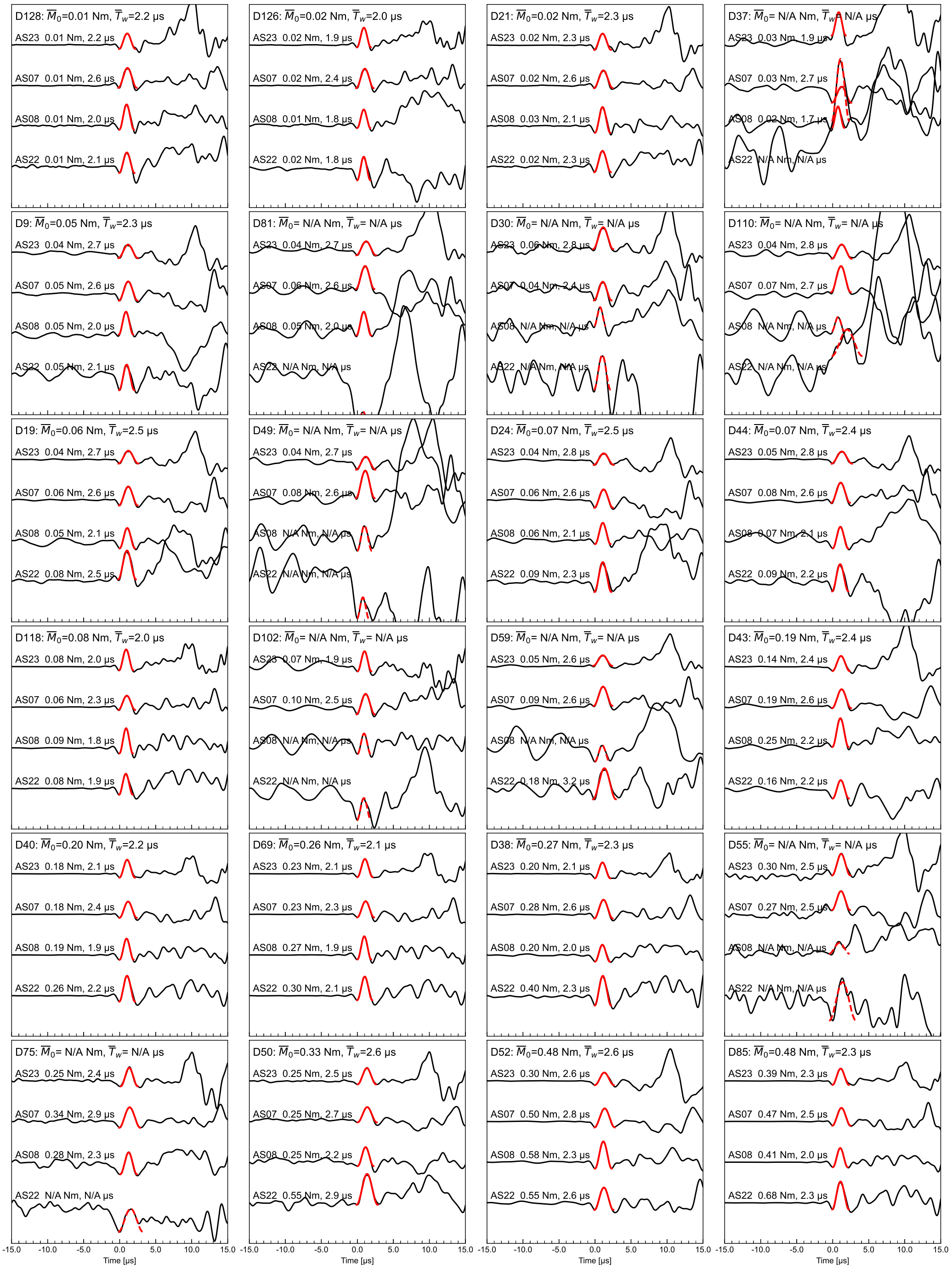

Fig. S26 Fitting the cosine STF with the attenuation-corrected observations to evaluate the source parameters of GP events, as shown in Fig. 3b of the main text. For visualization, the amplitudes of both the observed waveforms (black lines) and the best-fit cosine STFs (thick red lines) are normalized by the mean of the maximum values of the best-fit synthetic STF evaluated during the fitting process. A two-way low-pass filter with a cutoff at 1 MHz was applied before the attenuation correction. STF fittings that were excluded due to large residuals or low signal-to-noise ratios of the P-waveforms are indicated by red dashed lines. The mean seismic moment ($\overline{M}_0$) and source duration ($\overline{T}_w$) were calculated only when valid data from all four sensors were available.

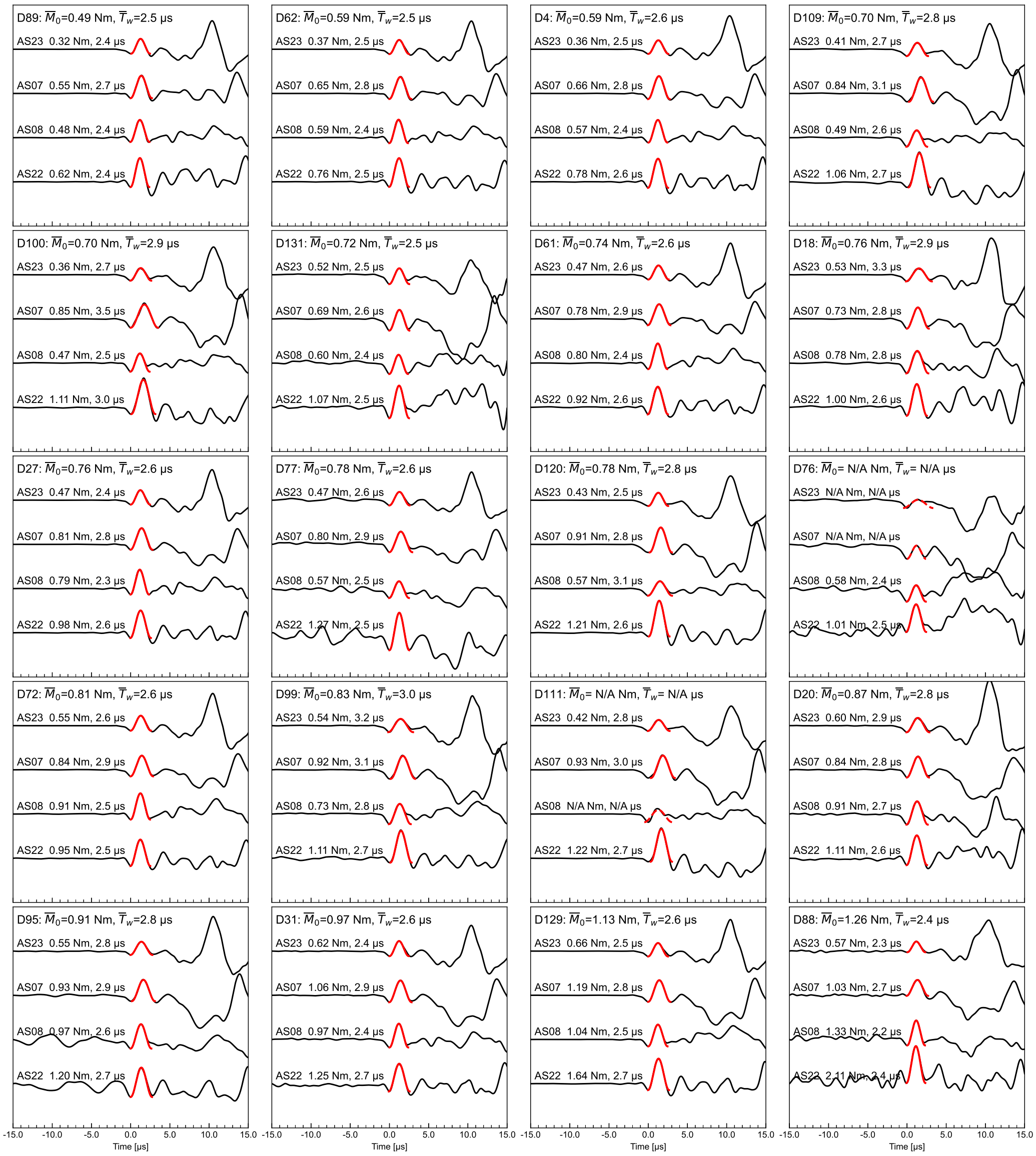

Fig. S26 (continued)

Supplementary References

Fukuyama E, Xu S, Yamashita F, et al. (2016) Cohesive Zone Length of Metagabbro at Supershear Rupture Velocity. J. Seismol. 20(4):1207–1215. https://doi.org/10.1007/s10950-016-9588-2

Herrmann RB (2013) Computer Programs in Seismology: an Evolving Tool for Instruction and Research. Seismol. Res. Lett. 84(6):1081–1088. https://doi.org/10.1785/0220110096

Kwiatek G, Charalampidou EM, Dresen G, et al. (2014) An Improved Method for Seismic Moment Tensor Inversion of Acoustic Emissions Through Assessment of Sensor Coupling and Sensitivity to Incidence Angle. Int. J. Rock Mech. Min. Sci. 65:153–161. https://doi.org/10.1016/j.ijrmms.2013.11.005

Ljung L (1987) System Identification: Theory for the User. Prentice-Hall, ISBN 9780138816407

McLaskey GC, Glaser SD (2010) Hertzian Impact: Experimental Study of the Force Pulse and Resulting Stress Waves. J. Acoust. Soc. Am. 128(3):1087–1096. https://doi.org/10.1121/1.3466847

McLaskey GC, Glaser SD (2012) Acoustic Emission Sensor Calibration for Absolute Source Measurements. J. Nondestruct. Eval. 31(2):157–168. https://doi.org/10.1007/s10921-012-0131-2

Miller R, McIntire P (1987) Nondestructive Testing Handbook Second Edition Vol. 5: Acoustic Emission Testing. Am. Soc. Nondestructive Testing, ISBN 9780931403026

SEED Reference Manual (2012) v2.4, accessed March 11, 2025, at URL http://www.fdsn.org/pdf/SEEDManual_V2.4.pdf

Watson G (1944) A Treatise on the Theory of Bessel Functions, 2Nd Ed. Cambridge Mathematical Library, Cambridge University Press, ISBN 9780521483919

Yamashita F, Fukuyama E, Xu S (2022) Foreshock Activity Promoted by Locally Elevated Loading Rate on a 4-m-long Laboratory Fault. J. Geophys. Res. 127(3):e2021JB023336. https://doi.org/10.1029/2021JB023336
